# Hepatoprotective Effects of Maillard Reaction Products From *Perinereis aibuhitensis*: Identification and Metabolomic Insights in a Mouse Model of Alcoholic Liver Disease

**DOI:** 10.1002/fsn3.72417

**Published:** 2026-10-07

**Authors:** Tonglei Qiao, Yuchun Lu, Teng Teng, Zichen Deng, Feng Liu, Chun‐E Liu

**Affiliations:** ^1^ Ocean College Yantai Institute of China Agricultural University Yantai Shandong China; ^2^ College of Food Science and Nutritional Engineering China Agricultural University Beijing China; ^3^ Shandong Key Laboratory of Digital Fishery Yantai Shandong China

**Keywords:** alcoholic liver disease, hepatoprotection, Maillard reaction, metabolomics, peptidomics, *Perinereis aibuhitensis*

## Abstract

Alcoholic liver disease (ALD) is a major global health burden with limited therapeutic options. The primary aim of this study was to investigate the hepatoprotective effects of Maillard reaction products derived from *Perinereis aibuhitensis* (P‐MRPs) in a mouse model of chronic alcohol‐induced liver injury. Male ICR mice were fed a Lieber–DeCarli alcoholic liquid diet for 6 weeks with or without P‐MRPs administration. P‐MRPs intervention significantly reduced serum alanine aminotransferase (ALT) and aspartate aminotransferase (AST) activities, alleviated hepatic histopathological injury, restored antioxidant enzyme activities (superoxide dismutase and glutathione peroxidase), and lowered malondialdehyde and interleukin (IL‐6, IL‐18, and IL‐1β) levels. Mechanistically, P‐MRPs treatment upregulated hepatic nuclear factor erythroid 2‐related factor 2 (NRF2), downregulated NF‐κB p65 and tumor necrosis factor (TNF)‐α, and restored carnitine O‐palmitoyltransferase 1A (CPT1A) expression. Liver metabolomic analysis revealed that P‐MRPs administration increased N‐acyl amino acids and tryptophan‐derived metabolites while reversing alcohol‐induced accumulation of glycerophospholipids and acylcarnitines. In vitro, P‐MRPs reduced ethanol‐induced ROS accumulation and attenuated ALT and AST leakage in AML12 hepatocytes. Compositional profiling identified 27 small‐molecule compounds with significant changes in P‐MRPs abundance, and a high proportion of small‐molecule peptides. Collectively, these findings suggest that P‐MRPs protect against ALD‐related liver injury, potentially through coordinated regulation of antioxidant, inflammatory, and lipid metabolic pathways, and provide a hypothesis‐generating framework for future mechanistic investigations.

## Graphical abstract

This graphical abstract summarizes the hepatoprotective effects of Maillard reaction products derived from *Perinereis aibuhitensis* (P‐MRPs) in alcohol‐induced liver injury. Male ICR mice were fed a Lieber‐DeCarli alcohol liquid diet (1%–5%, w/v) for 6 weeks with or without P‐MRPs treatment. P‐MRPs improved liver function and histopathology, alleviated oxidative stress, suppressed inflammation, and improved lipid metabolism. Metabolomics revealed increased beneficial metabolites and decreased harmful metabolites, indicating multi‐pathway hepatoprotection.

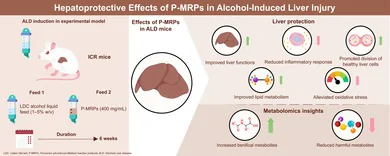

## 1 Introduction

Alcoholic liver disease (ALD) is a major global public health concern (Park and Seo 2025). Excessive alcohol consumption adversely affects hepatic metabolism, leading to the accumulation of toxic intermediates such as acetaldehyde and other ethanol oxidation products. These metabolites disrupt intracellular signaling pathways, impair transcriptional regulation, and induce oxidative stress, lipid deposition, and defective autophagy, ultimately progressing to alcoholic hepatitis, steatosis, and hepatocellular carcinoma (Brahadeeswaran et al. 2023). Despite various treatment strategies, such as alcohol abstinence, pharmacological intervention, and surgical treatments, the efficacy of ALD clinical management remains suboptimal owing to poor abstinence adherence, delayed diagnosis and treatment initiation, lack of targeted drugs, and complex disease heterogeneity (Dixon et al. 2025; Yang et al. 2025). Furthermore, the frequent occurrence of malnutrition among patients with ALD further exacerbates disease progression and complications (Styskel et al. 2019). Clinical interventions, such as corticosteroid therapy, nutritional modulation, and dietary interventions to mitigate alcohol‐induced liver injury, have recently garnered much attention (Shasthry and Sarin 2016). In particular, food‐derived bioactive compounds with multitarget effects, low toxicity, and hepatoprotective potential have emerged as promising alternatives for preventing and managing ALD (Ding et al. 2022).

*Perinereis aibuhitensis* is a marine benthic annelid widely distributed in intertidal zones of Russia, Japan, and the Yellow–Bohai Sea region of China. It is a rich source of essential fatty acids and trace elements, with an amino acid profile similar to that of the human body (Li et al. 2016; Liu et al. 2022), highlighting its potential for functional food applications. Enzymatic hydrolysates derived from *P. aibuhitensis* demonstrate diverse bioactivities, including antioxidant, antitumor, and antithrombotic properties (Chen and Wang 2017; Li et al. 2017; Song et al. 2024; Teng et al. 2025). However, its poor sensory characteristics hinder its broader application in nutraceutical formulations (Li et al. 2016) despite increased production volumes (Yang et al. 2015).

The Maillard reaction, a common non‐enzymatic browning process in food processing, enhances the aroma and flavor profiles of protein hydrolysates derived from marine and aquatic sources (Long et al. 2025; Tang et al. 2025; Zhao et al. 2024). Maillard reaction products (MRPs) offer antioxidant benefits and enhance sensory qualities of functional ingredients, thereby increasing their acceptance and utility in food systems (Dou et al. 2024; He et al. 2020). Despite these benefits, the biofunctional potential of MRPs derived from *P. aibuhitensis* (P‐MRPs), especially in liver health, remains largely unexplored, with existing studies focusing narrowly on flavor and aroma (Guo et al. 2012; Teng et al. 2026).

In this study, we investigated the protective effects of P‐MRPs in a murine model of alcohol‐induced liver injury, focusing on three key pathological processes central to ALD progression: oxidative stress, hepatic inflammation, and lipid dysregulation (Hong et al. 2024; Zhou et al. 2026). We assessed physiological, biochemical, and histopathological parameters, and performed untargeted metabolomics to identify metabolic pathways affected by P‐MRPs. To link metabolomic alterations with phenotypic outcomes at the molecular level, we measured hepatic protein levels of key regulatory factors involved in antioxidant defense (nuclear factor erythroid 2‐related factor 2 [NRF2]), inflammatory signaling (NF‐κB p65/tumor necrosis factor [TNF]‐α), and lipid metabolism (carnitine O‐palmitoyltransferase 1A [CPT1A]). We further conducted in vitro experiments using AML12 hepatocytes to evaluate the direct antioxidant and cytoprotective effects of P‐MRPs at the cellular level. We also used metabolomic and peptidomic profiling to preliminarily characterize the chemical and peptidic composition of P‐MRPs. Collectively, these analyses demonstrate that P‐MRPs exert hepatoprotective effects in ALD mice and generate testable hypotheses for future mechanistic studies, although their translational potential requires further investigation.

## 2 Methods

### 2.1 Reagents

*P. aibuhitensis* (a single batch, artificial feeding time, 5–6 months) was sourced commercially from Shandong Dongying Zhenyu Aquatic Resources Development Co. Ltd. (Shandong, China). The mouse normal hepatocyte cell line (AML12; CL‐0602) and AML12 cell complete medium (CM‐0602) were obtained from Wuhan Pricella Biotechnology Co. Ltd. (Hubei, China). Food‐grade flavor proteases (50,000 U/g) and neutral proteases (20,000 U/g) were supplied by Shandong Qilu Biotechnology Group Co. Ltd. (Shandong, China). L‐cysteine, glucose, and D‐ribose, all of food‐grade purity, were acquired from Shenzhen Xinrong Biotechnology Co. Ltd. (Guangdong, China), while food‐grade alcohol (95%) was sourced from Hanyong Alcohol Co. Ltd. (Henan, China). The Lieber‐DeCarli (LDC) alcoholic liquid diet and its matching control diet were obtained from Daitz Biotechnology Co. Ltd. (Jiangsu, China). Commercial assay kits for superoxide dismutase (SOD; A001‐3‐2), glutathione peroxidase (GPx; A005‐1‐2), alanine aminotransferase (ALT; C009‐2‐1), aspartate aminotransferase (AST; C010‐2‐1), malondialdehyde (MDA; A003‐1‐2), total cholesterol (TC; A111‐1‐1), and triglyceride (TG; A110‐1‐1) were purchased from Nanjing Jiancheng Bioengineering Institute (Nanjing, China). A reactive oxygen species (ROS) kit was obtained from Shanghai Beyotime Biotechnology Co. Ltd. (Shanghai, China). Enzyme‐linked immunosorbent assay (ELISA) kits for interleukin (IL)‐1β (MM‐0040M1), IL‐6 (MM‐0163M1), IL‐18 (MM‐0169M1), and tumor necrosis factor (TNF)‐α (MM‐0132M1) were procured from Meimian Industrial Co. Ltd. (Jiangsu, China); kits for nuclear factor kappa‐B (NF‐κB) p65 (RE3044M) and NRF2 (RE3062M) were obtained from Reed Biotechnology Co. Ltd. (Hubei, China); and a kit for CPT1A (CSB‐EL005922MO) was purchased from Cusabio Technology Co. Ltd. (Hubei, China). Unless otherwise indicated, all experimental procedures followed the manufacturers' protocols. All other chemicals were of analytical grade.

### 2.2 Preparation of P‐MRPs

Based on previous research in our laboratory, we used the RSM‐BP‐GA model to prepare *P. aibuhitensis*‐derived enzyme hydrolysates with the highest degree of hydrolysis (PEH‐HDH) (Teng et al. 2025). Fresh *P. aibuhitensis* specimens were purchased from Shandong Dongying Zhenyu Aquatic Resources Development Co. Ltd. (Shandong, China). The samples were homogenized, distilled water was added at a solid‐to‐liquid ratio of 1:2.5, and ultrasound pretreatment was performed using a sonicator at 390 W and 30 kHz for 31 min. Enzymatic hydrolysis was performed by adding 970 U/g of a composite hydrolytic protease (neutral protease: flavor protease = 1:1) at 50°C and pH 7.0. The total hydrolysis time was 3 h, after which the enzyme was deactivated by boiling the hydrolysate for 5 min. The mixture was then filtered through a 40‐mesh gauze to obtain the enzymatic hydrolysate of *P. aibuhitensis*. Subsequently, 60 g of the enzymatic hydrolysate was mixed with 2.5% (w/w) L‐cysteine and 3.21% reducing sugar (mass fraction, ribose: glucose = 1:1). The Maillard reaction was conducted at 121°C, pH 6.5, for 64 min. After the reaction, the solution was diluted 50‐fold and 10‐fold for absorbance measurements at 294 nm and 420 nm, respectively. P‐MRPs were accepted when they met the following criteria: A_294_ ≥ 0.60 and A_420_ ≥ 0.25. We previously characterized their volatile flavor compounds and free amino acid composition in our published study on flavor and bioactivity profiles (Teng et al. 2026).

### 2.3 Cell Culture Experiments

AML12 mouse hepatocytes in logarithmic growth phase were cultured at 37°C in a humidified atmosphere containing 5% CO_2_, digested with 0.25% trypsin, centrifuged, and resuspended at 2 × 10^5^ cells/mL according to Yang et al. (2020). Cell suspensions were seeded into 96‐well plates at 100 μL per well. After complete adhesion, cells were assigned to the Alcohol, PEH‐HDH (enzyme hydrolysates of *P. aibuhitensis* with the highest degree of hydrolysis), and P‐MRPs (*P. aibuhitensis*‐derived Maillard reaction products) groups. Six concentration gradients were established for each group, with six replicate wells per concentration. Ethanol was applied at 0, 31.25, 62.5, 125, 250, and 500 mmol/L, whereas PEH‐HDH and P‐MRPs were applied at 0, 50, 100, 200, 400, and 800 mg/mL. After treatment with fresh medium containing the indicated concentrations of ethanol or test substances for 24 h, 10 μL of CCK‐8 solution was added to each well, and plates were incubated at 37°C in the dark for 3 h. Absorbance was measured at 450 nm, and cell viability was calculated to determine the optimal ethanol and treatment concentrations. Based on the viability curves, 250 mmol/L ethanol was selected as the modeling concentration, and 100 mg/mL PEH‐HDH or P‐MRPs was used for subsequent experiments.

Following Sun et al. (2024), AML12 cells were seeded into 96‐well plates. After adhesion, the cells were divided into four groups: Control (complete medium without ethanol or test substances), Model/Alcohol group (medium containing 250 mmol/L ethanol), PEH‐HDH group (medium containing 250 mmol/L ethanol and 100 mg/mL PEH‐HDH), and P‐MRPs group (medium containing 250 mmol/L ethanol and 100 mg/mL P‐MRPs). After 24 h of co‐incubation with ethanol and the indicated test substances, ALT, AST, and ROS levels in the cell supernatants were measured according to the manufacturers' instructions for the respective assay kits. All experiments were independently repeated six times.

### 2.4 Experimental Animals

All animal experiments were conducted in compliance with the guidelines of the Animal Ethics Committee of China Agricultural University and Suzhou Weiyuan Biotechnology Co. Ltd., the ARRIVE guidelines, and the National Research Committee guidelines for the care and use of experimental animals (approval number: IACUC‐20240929‐001).

Specific pathogen‐free (SPF) ICR male mice (6 weeks old, weight 20.9 ± 0.2 g) were purchased from Jiangsu Huachuang Xinnuo Pharmaceutical Technology Co. Ltd. (license No.: SCXK [Jiangsu] 2020–0009). Mice were housed in an SPF facility under controlled conditions (temperature 20°C–25°C, relative humidity 50%–60%, and 12 h light/dark cycle). Following 1 week of acclimatization, mice were randomly assigned to three groups (*n* = 6/group): liquid‐feed control (C), alcohol model (AS), and P‐MRPs intervention (PP). For PP treatment, lyophilized P‐MRPs were reconstituted in distilled water at 400 mg/mL and administered by gavage at 20 mL/kg body weight. The concentration and dose were determined from in vitro AML12 mouse hepatocyte experiments (see Section 2.3), a simplified IVIVE (in vitro–in vivo extrapolation) calculation, and the maximum tolerable gavage volume for mice (Umehara and Camenisch 2012; Zhang et al. 2018). Except for the control group, a transitional feeding strategy was implemented during the acclimatization week, gradually increasing ethanol concentration from 1% to 5% (w/v). Alcoholic liver injury was induced, and the model was established using an LDC alcoholic liquid diet administered via a specialized feeder without supplementary drinking water, following the protocol described by Wang, Shi, et al. (2022); Wang, Yu, et al. (2022). After the adaptation period, the C group was fed an isocaloric LDC control diet; however, the other groups were fed an LDC alcohol liquid diet for 6 weeks. Both diets had a caloric density of 1 kcal/mL. Water or P‐MRPs were administered by gavage at regular intervals every day, and the physical condition and activity were monitored daily throughout the experiment. At the end of the experiment, after a 12 h fasting period, the mice were euthanized using bone‐dry carbon dioxide (purity > 99.9%) (Stuckey et al. 2023). The CO_2_ displacement rate was 60% of the euthanasia container (27 × 25 × 20 cm) volume per min (8.10 L/min). Blood samples and whole livers were then collected for subsequent analyses.

### 2.5 Determination of Body Weight and Liver Index

The body weight of each mouse was recorded before (BWB, g) and after (BWA, g) the experiment. Following euthanasia, the entire liver of the mice was excised, weighed, and recorded as liver weight (LW, g). The liver index (LI, %) was calculated using the following formula (Equation 1):

(1) $$\mathrm{LI}\;\left(\%\right)=\frac{\mathrm{LW}}{\mathrm{BWA}}\times 100$$

Eighteen mice were analyzed, with six biological replicates per group.

### 2.6 Serum Biochemical Index Analysis

Blood samples were centrifuged at 1000 × *g* for 15 min at 4°C to obtain serum, as previously described (Wang, Yu, et al. 2022). Commercial assay kits were used to measure AST and ALT activities, as well as TC and TG levels in mouse serum. Eighteen mice were analyzed, with six replicates per group.

### 2.7 Hepatic Histopathology and Biochemical Index Analysis

After morphological observation and size measurement, mouse liver tissues were fixed in 4% paraformaldehyde for 24 h, dehydrated, paraffin‐embedded, and sectioned at a 5 μm thickness (Wang, Shi, et al. 2022; Wang, Yu, et al. 2022). The sections were then dewaxed with xylene, rehydrated with an alcohol gradient, and stained with hematoxylin and eosin (H&E). Histological changes were observed under a light microscope. Commercial assay kits were used to measure antioxidant enzyme biomarkers (SOD and GPx) and oxidative stress marker (MDA) in liver tissues. ELISA kits were used to detect the levels of transcription factors (NRF2 and NF‐κB p65), inflammatory cytokines (TNF‐α, IL‐6, IL‐18, and IL‐1β), and metabolic enzyme (CPT1A). Eighteen mice were analyzed, with six biological replicates per group.

### 2.8 Metabolomic Analysis of Liver Tissues

Liver tissue samples from 12 mice (*n* = 4 per group) were prepared for metabolomic profiling. Approximately 100 mg of tissue was pulverized in liquid nitrogen using a mortar and pestle. The resulting powder was homogenized in an ice‐cold 80% (v/v) methanol solution, vortexed thoroughly, and incubated on ice for 5 min to precipitate proteins. After an initial centrifugation (15,000 × *g*, 20 min, 4°C), the supernatant was diluted with liquid chromatography–mass spectrometry (LC–MS) grade water to a final methanol concentration of 53% (v/v). A second centrifugation step under identical conditions was performed, and the clarified supernatant was stored at −80°C until analysis.

Ultra‐high‐performance liquid chromatography–mass spectrometry (UHPLC–MS) was carried out by Novogene Co. Ltd. (Beijing, China) on a Vanquish UHPLC system coupled to an Orbitrap Q Exactive HF mass spectrometer (Thermo Fisher Scientific). Separation was achieved on a Hypersil Gold column (100 × 2.1 mm, 1.9 μm) at a flow rate of 0.2 mL/min over a 17‐min linear gradient, using 0.1% formic acid in water (A) and methanol (B) as mobile phases. Mass detection was performed in positive electrospray ionization mode with the following settings: spray voltage, 3.5 kV; capillary temperature, 320°C; sheath gas, 35 psi; auxiliary gas, 10 L/min at 350°C; S‐lens RF, 60.

Quality control (QC) samples, prepared by pooling equal volumes of all liver tissue extracts, were injected every six samples throughout the LC–MS/MS sequence to monitor instrument stability and analytical reproducibility.

Raw data were processed using Compound Discoverer 3.3 (Thermo Fisher Scientific) for peak alignment, extraction, and quantification. Peak areas were normalized to the first QC sample, with a mass tolerance of 5 ppm and a signal intensity threshold of 30%. Total ion current normalization was applied, and molecular formulas were predicted based on adduct, molecular, and fragment ion patterns. Final compound identification was accomplished by querying the mzCloud, mzVault, and MassList databases.

For quality assurance, metabolites with a coefficient of variation (CV) > 30% across QC samples were excluded from subsequent analysis. Pairwise Pearson correlation coefficients were then calculated among QC samples using the relative quantitative values of detected metabolites. The consistently high QC inter‐correlations (*r* > 0.99, Figure S1) confirmed the stability and reproducibility of the analytical platform.

For data preprocessing, raw MS files were converted to mzXML format using ProteoWizard and processed in XCMS for peak detection, alignment, and retention‐time correction. Peak features missing in more than 50% of samples within any group were excluded, and remaining missing values were imputed using the half‐minimum method, in which each missing value was replaced with half of the minimum positive value detected for that metabolite across all samples.

To control the false discovery rate (FDR) during multiple hypothesis testing, raw metabolomics *p‐*values were adjusted using the Benjamini–Hochberg (BH) method. Metabolites were considered significant when the FDR‐adjusted *q*‐value was < 0.05.

Statistical analysis and data processing were conducted using Python (v2.7.6), R (v3.4.3), and the CentOS 6.6 operating system. Metabolites displaying a CV exceeding 30% in QC samples were removed. Subsequent processing was performed with the MetaX software (BGI Tech). Principal component analysis (PCA) provided variable importance in projection (VIP) scores. Univariate analysis (Student's *t*‐test) identified metabolites with significant abundance changes, and fold change (FC) values were calculated. Differentially abundant metabolites (VIP > 1, *p* < 0.05, FC > 2 or FC < 0.5, and FDR‐adjusted *q* < 0.05) were annotated against the HMDB, Kyoto Encyclopedia of Genes and Genomes (KEGG), and LIPID MAPS databases. Data visualization (pie charts, upset plots, heatmaps) was performed using Origin 2021 and R. Before analysis, metabolite intensities were log_10_‐transformed.

### 2.9 Compositional Characterization of P‐MRPs

Small‐molecule compounds in PEH‐HDH and P‐MRPs were characterized by LC–MS according to a previously reported protocol (Dias et al. 2016). Briefly, 100 μL of each sample was mixed with 300 μL of methanol, centrifuged at 15,000 × *g* for 20 min, and the supernatant was analyzed by UHPLC–MS/MS. Chromatographic separation was performed on an ACQUITY Premier HSS T3 column (2.1 × 100 mm, 1.8 μm) at 45°C using 0.1% formic acid in water (A) and methanol (B) as mobile phases at a flow rate of 0.3 mL/min over a 16‐min gradient. Mass spectrometry was performed on a Q Exactive‐PLUS instrument in positive ion mode. Full‐scan MS^1^ spectra were acquired at a resolution of 70,000 over an m/z range of 150–1500, and data‐dependent MS/MS fragmentation of the top ten precursor ions was conducted at a resolution of 17,500 using stepped normalized collision energy (NCE) values of 10, 30, and 55. Raw data were processed using MS‐DIAL 4.70 for peak picking, deconvolution, and alignment.

Small‐molecule compounds were annotated by searching the MassBank, ReSpect, and GNPS databases (14,951 total records) using the following criteria: retention time (RT) tolerance ≤ 0.05 min, MS^1^ tolerance ≤ 0.01 Da, MS^2^ tolerance ≤ 0.05 Da, and identification score ≥ 80. Only compounds with a total score ≥ 60 were retained, and those with a CV > 30% in QC samples were excluded. All reported compounds were putatively annotated at MSI Level 2. Univariate analysis using Student's *t*‐test was performed to identify small‐molecule compounds with significant abundance changes (*p* < 0.05, FC > 2 or FC < 0.5, and FDR‐adjusted *q* < 0.05).

Peptidomic profiling was performed to quantify peptides in P‐MRPs, with all analyses conducted in triplicate. Peptides were initially enriched using molecular‐weight cutoff filtration. Specifically, 1000 μL of each sample was subjected to ultrafiltration using a 10‐kDa molecular‐weight‐cutoff device, followed by centrifugation at 12,000 × *g* for 10 min to collect the < 10‐kDa filtrate.

For subsequent processing, a 200‐μL aliquot of the enriched peptide solution was reduced with 2 μL of 1 M dithiothreitol (DTT; final concentration, 10 mM) at 56°C for 1 h and alkylated in the dark with 4 μL of 1 M iodoacetamide (IAM; final concentration, 20 mM) at 25°C for 40 min. Residual IAM was quenched by adding an additional 2 μL of 1 M DTT (final concentration, 10 mM). Desalting was performed using SP2 magnetic beads. Processed peptides, estimated at 20 μg from a 50 μg protein‐equivalent digest, were reconstituted in 15 μL of 0.1% formic acid, mixed with 10 μL of pre‐equilibrated beads, and gently vortexed. After adding 190 μL of 100% acetonitrile to achieve a final concentration of 95% and incubating for 5 min, the supernatant was removed following magnetic separation. The beads were washed three times with 200 μL of 100% acetonitrile for 2 min per wash, and peptides were eluted twice with 54 μL of 2% acetonitrile. The combined eluates were vacuum‐dried and stored until LC–MS/MS analysis.

Chromatographic separation was performed using a pre‐column (150 μm × 50 mm, Reprosil‐Pur 120 C18‐AQ, 3 μm) coupled to an analytical column (150 μm × 170 mm, Reprosil‐Pur 120 C18‐AQ, 1.9 μm). The mobile phases comprised 0.1% aqueous formic acid (phase A) and 0.1% formic acid in 80% acetonitrile (phase B), delivered at 600 nL/min. The gradient program was as follows: 4%–8% B from 0 to 2 min, 8%–28% B from 2 to 35 min, 28%–40% B from 35 to 55 min, 40%–95% B from 55 to 56 min, and 95% B from 56 to 66 min. Mass spectrometry was conducted in data‐dependent acquisition mode. Full‐scan MS spectra (m/z 100–1500) were acquired at a resolution of 70,000, with an AGC (Automatic Gain Control) target of 3 × 10^6^ and a maximum injection time of 100 ms. The 20 most intense precursor ions were selected for fragmentation at an NCE of 28%. MS/MS spectra were acquired at a resolution of 17,500, with an AGC target of 1 × 10^5^ and a maximum injection time of 50 ms. Raw data were processed using Proteome Discoverer against the Nereididae database. Search parameters included carbamidomethylation as a fixed modification; methionine oxidation and N‐terminal acetylation as variable modifications; unspecific enzyme cleavage; and mass tolerances of 20 ppm for MS and 0.02 Da for MS/MS.

We generated graphical representations using R v3.4.3 and Origin 2021. Peptide instability indices were calculated using the ExPASy ProtParam online tool. Before analysis, peptide data were log10‐transformed and Z‐score standardized, and missing values were imputed using the half‐minimum method.

### 2.10 Statistical Analysis

All data are expressed as the mean ± standard error of the mean (SEM). Statistical evaluation was conducted using SPSS software (version 27.0; SPSS Inc., Chicago, IL, USA). Group differences were assessed using a one‐way analysis of variance, with post hoc comparisons performed using Tukey's test; a probability (*p*) value of less than 0.05 was considered statistically significant. Furthermore, all graphical representations of the data were created using R and Origin (Origin 2021 version).

## 3 Results

### 3.1 Effects of P‐MRPs on Alcohol‐Induced Liver Injury

No significant differences in BWB were observed among the three groups at baseline (Table 1). After the 6‐week feeding period, BWA was significantly lower in the AS group than in the C group (*p* < 0.05), whereas LW and LI were significantly higher (*p* < 0.05). In the PP group, BWA, LW, and LI did not differ significantly from those in the C group, but all three parameters differed significantly from those in the AS group (*p* < 0.05; Table 1).

**TABLE 1:** Body weight, liver weight, and liver index of different mouse groups.

Indicator name	C	AS	PP
BWB (g)	20.72 ± 0.64^a^	20.75 ± 0.62^a^	20.83 ± 0.59^a^
BWA (g)	24.71 ± 0.53^a^	21.63 ± 0.87^b^	23.95 ± 0.78^a^
LW (g)	0.94 ± 0.05^b^	1.18 ± 0.07^a^	0.98 ± 0.05^b^
LI (%)	3.79 ± 0.18^b^	5.44 ± 0.23^a^	4.10 ± 0.23^b^

*Note:* *n* = 6 per group; different lowercase letters within the same row indicate significant differences (*p* < 0.05); the same or no letter indicates a non‐significant difference (*p* > 0.05).

Abbreviations: AS, alcohol model group; BWA, weight of mice after the experiment; BWB, weight of mice before the experiment; C, control group; LI, liver index; LW, weight of the entire liver of mice; PP, P‐MRPs intervention group.

Livers from the C group exhibited a normal dark red appearance (Figure 1A). In contrast, livers from the AS group were visibly enlarged and discolored, with apparent morphological alterations. Livers from the PP group were similar in appearance to those from the C group, showing reduced enlargement and improved coloration.

**FIGURE 1:**
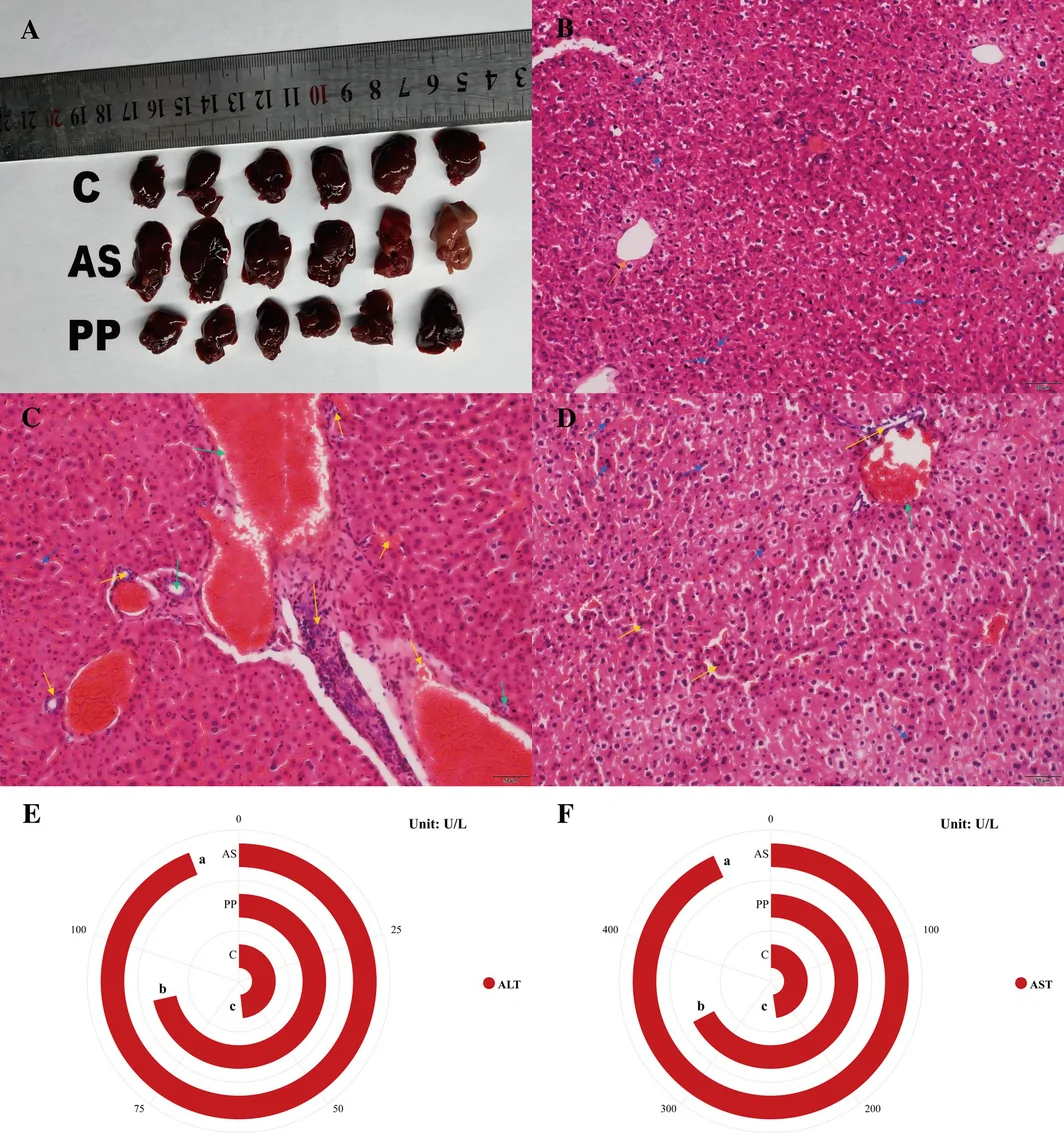
P‐MRPs attenuate alcohol‐induced liver injury in mice. (A) Representative liver photographs from the C (control), AS (alcohol model), and PP (P‐MRPs intervention) groups. (B–D) Representative H&E‐stained liver sections from the C (B), AS (C), and PP (D) groups. Yellow arrows: Inflammatory infiltration; green arrows: Hepatic blood vessels; blue arrows: Binucleate hepatocytes; orange arrows: Portal vein. Scale bar = 50 μm. Three histological sections were evaluated per sample. (E, F) Serum ALT (E) and AST (F) activities. *n* = 6 per group; lowercase letters above bars within the same panel denote significant differences (*p* < 0.05; one‐way ANOVA with Tukey's multiple comparison test). P‐MRPs: *P. aibuhitensis*‐derived Maillard reaction products. AST, Aspartate aminotransferase; ALT, Alanine aminotransferase.

H&E staining revealed normal hepatic architecture in the C group, characterized by well‐organized hepatic cords and abundant binucleate hepatocytes (Figure 1B). In the AS group, extensive inflammatory infiltration, focal hemorrhage, and a marked reduction in binucleate hepatocytes were observed (Figure 1C). Compared with the AS group, the PP group showed reduced inflammatory infiltration and increased numbers of binucleate hepatocytes (Figure 1D).

Serum ALT and AST activities, established indicators of hepatocellular injury, were significantly elevated in the AS group compared with the C group. P‐MRPs intervention significantly reduced both ALT and AST activities compared with the AS group (*p* < 0.05; Figure 1E–F).

### 3.2 Effects of P‐MRPs on Hepatic Oxidative Stress

To assess oxidative stress, we measured NRF2 protein levels, MDA levels, and SOD and GPx activities in the three groups (Figure 2A–D). Hepatic NRF2 protein levels were significantly lower in the AS group than in the C group (*p* < 0.05; Figure 2A) and significantly higher in the PP group than in the AS group (*p* < 0.05). MDA levels were significantly elevated in the AS group compared with the C group (*p* < 0.05) and significantly reduced in the PP group compared with the AS group (*p* < 0.05; Figure 2B). In contrast, SOD and GPx activities were significantly lower in the AS group relative to the C group (*p* < 0.05), and significantly higher in the PP group compared with the AS group (*p* < 0.05; Figure 2C,D).

**FIGURE 2:**
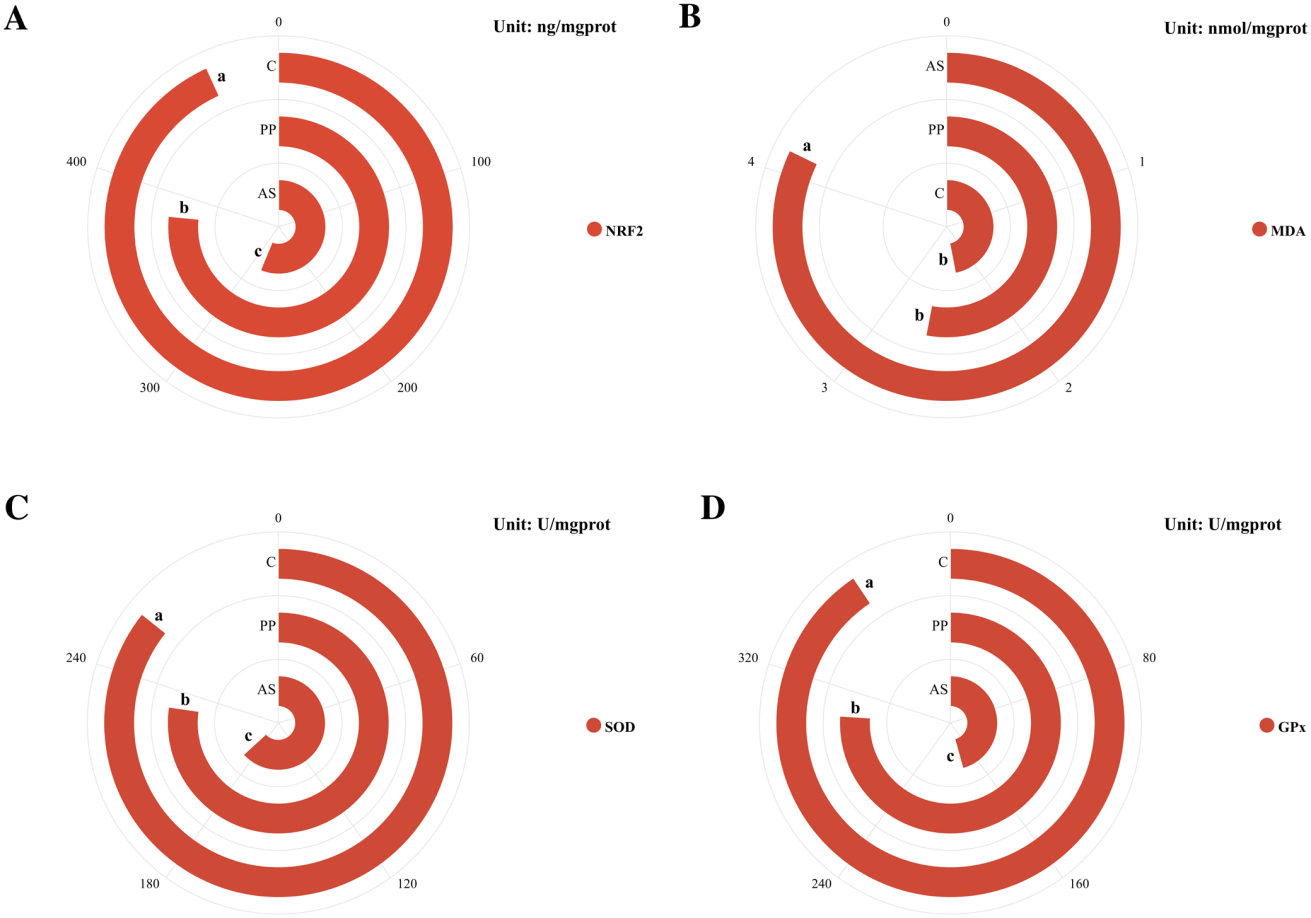
P‐MRPs reduce hepatic oxidative stress in mice. Hepatic (A) NRF2 levels, (B) MDA levels, (C) SOD activity, and (D) GPx activity. *n* = 6 per group; lowercase letters above bars within the same panel indicate significant differences (*p* < 0.05; one‐way ANOVA with Tukey's multiple comparison test). AS, alcohol model; C, control; GPx, glutathione peroxidase; MDA, Malondialdehyde; P‐MRPs, *P. aibuhitensis*‐derived Maillard reaction products; PP, P‐MRPs intervention; SOD, superoxide dismutase.

### 3.3 P‐MRPs and Hepatic Inflammation

To evaluate the effects of P‐MRPs on alcohol‐induced hepatic inflammation, hepatic protein levels of NF‐κB p65 and the pro‐inflammatory cytokines TNF‐α, IL‐6, IL‐1β, and IL‐18 were measured (Figure 3A–E). NF‐κB p65 levels were significantly higher in the AS group than in the C group (*p* < 0.05) and significantly lower in the PP group than in the AS group (*p* < 0.05; Figure 3A). Similarly, TNF‐α, IL‐6, IL‐1β, and IL‐18 levels were significantly increased in the AS group compared with the C group (*p* < 0.05) and significantly decreased in the PP group compared with the AS group (*p* < 0.05; Figure 3B–E).

**FIGURE 3:**
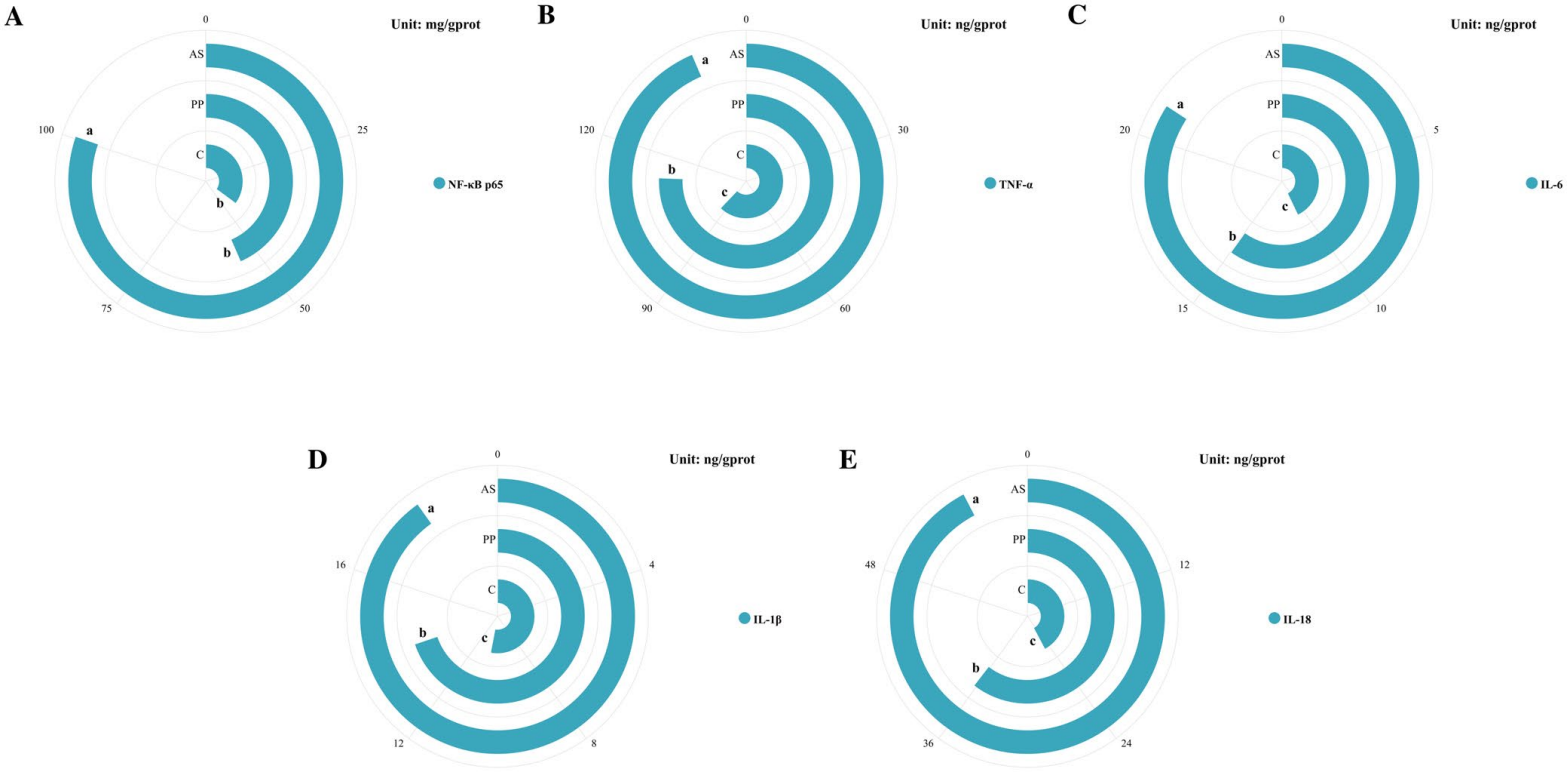
P‐MRPs suppress hepatic inflammation in mice. Hepatic (A) NF‐κB p65, (B) TNF‐α, (C) IL‐6, (D) IL‐1β, and (E) IL‐18 levels. *n* = 6 per group; lowercase letters above bars within the same panel denote significant differences (*p* < 0.05; one‐way ANOVA with Tukey's multiple comparison test). AS, alcohol model; C, control; IL, Interleukin; P‐MRPs: *P. aibuhitensis*‐derived Maillard reaction products; PP, P‐MRPs intervention; TNF‐α, Tumor necrosis factor‐alpha.

### 3.4 Effects of P‐MRPs on Hepatic Lipid Metabolism

Hepatic CPT1A protein levels and serum TC and TG levels were measured to assess the effects of P‐MRPs on lipid metabolism (Figure 4A–C). Hepatic CPT1A levels were significantly lower in the AS group than in the C group (*p* < 0.05; Figure 4A) and significantly higher in the PP group than in the AS group (*p* < 0.05). Serum TC and TG levels were significantly elevated in the AS group compared with the C group (*p* < 0.05) and significantly reduced in the PP group compared with the AS group (*p* < 0.05; Figure 4B,C).

**FIGURE 4:**
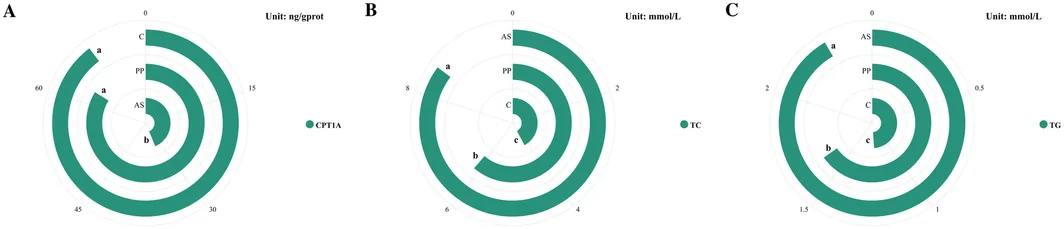
P‐MRPs improve hepatic lipid metabolism in mice. (A) Hepatic CPT1A, (B) serum TC, (C) serum TG. *n* = 6 per group; lowercase letters above bars within the same panel denote significant differences (*p* < 0.05; one‐way ANOVA with Tukey's multiple comparison test). P‐MRPs: *P. aibuhitensis*‐derived Maillard reaction products. AS, alcohol model; C, control; PP, P‐MRPs intervention; TC, total cholesterol; TG, triglyceride.

### 3.5 Effects of P‐MRPs on the Liver Metabolome

PCA showed clear differences in liver metabolite profiles among groups. PC1 and PC2 accounted for 47.17% and 15.81% of the total variation, respectively (Figure 5A). Using VIP > 1.0, FC > 2 or < 0.5, *p* < 0.05, and FDR‐adjusted *q* < 0.05 as screening criteria, 91 differential metabolites were identified in AS vs. C, and 89 were identified in PP vs. AS. In AS vs. C, 40 metabolites increased, and 51 decreased in the AS group. In PP vs. AS, 39 metabolites increased, and 50 decreased in the PP group.

**FIGURE 5:**
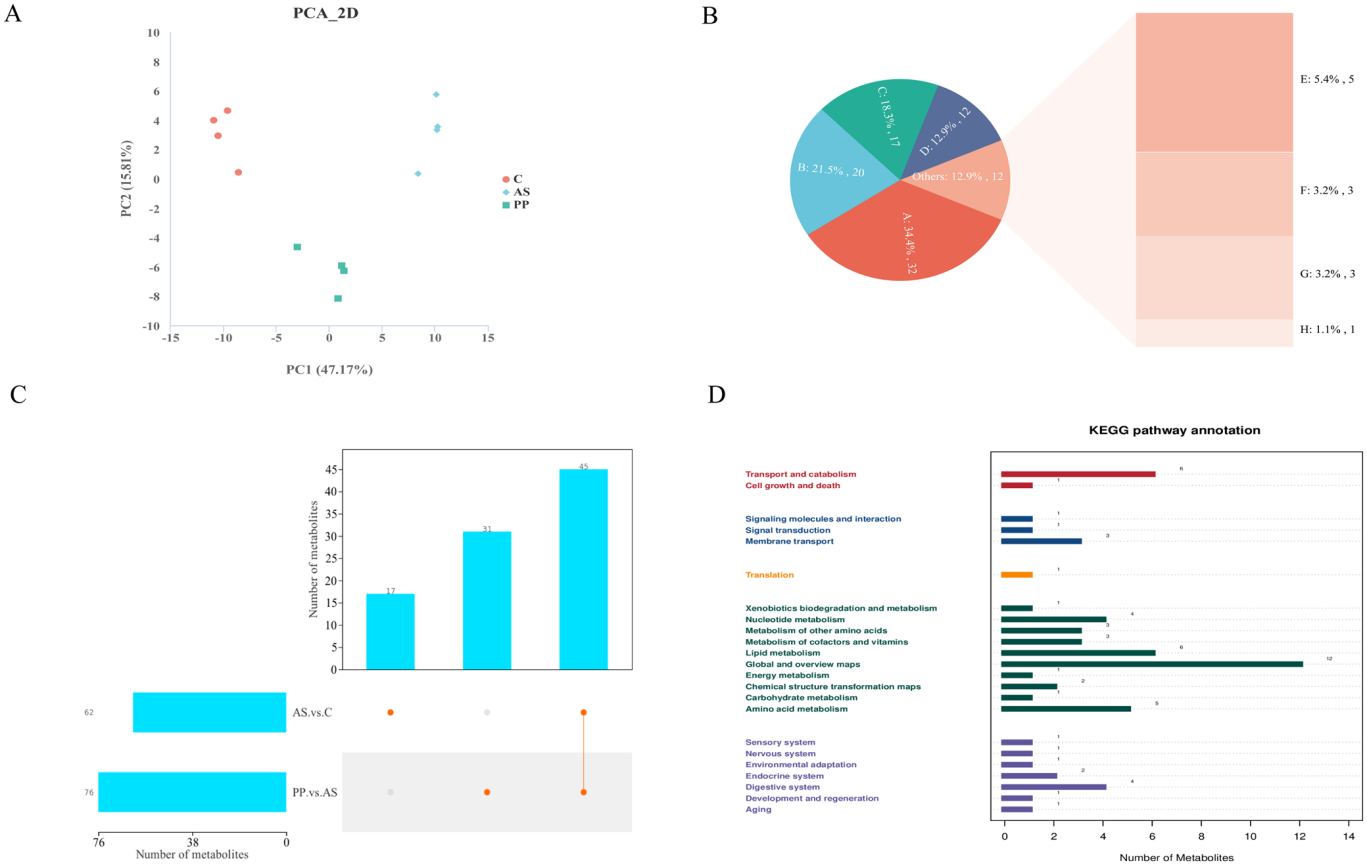
P‐MRPs impact hepatic tissue metabolites in mice (*n* = 4 per group). (A) PCA plot based on the liver metabolites of mice. The abscissa, PC1, and the ordinate, PC2, represent the scores of the principal components ranking first and second, respectively. The scattered dots of different colors represent the samples of different experimental groups. Samples with similar metabolite compositions tend to cluster. (B) Number and categories of differential metabolites identified by HMDB, KEGG, and LIPID MAPS. Categories A–H represent N‐acyl amino acids; other amino acids, peptides, and analogs; lipids and lipid‐like molecules; organoheterocyclic compounds; nucleosides, nucleotides, and analogs; organic acids and derivatives; organic oxygen compounds; and organic nitrogen compounds, respectively. (C) UpSet plot displaying shared differential metabolites between PP and AS and AS and C groups. (D) KEGG pathway annotation results. AS, Alcohol model; C, Control; HMDB, Human Metabolome Database; KEGG, Kyoto Encyclopedia of Genes and Genomes; LIPID MAPS, Lipid Metabolites and Pathways Strategy; PCA, Principal component analysis; P‐MRPs, *P. aibuhitensis*‐derived Maillard reaction products; PP, P‐MRPs intervention.

Integrated annotation using HMDB, KEGG, and Lipid Metabolites and Pathways Strategy (LIPID MAPS) identified 93 significantly differential metabolites (Figure 5B). N‐acyl amino acids were the most abundant class (34.4%), followed by other amino acids, peptides, and analogs (21.5%), lipids and lipid‐like molecules (18.3%), and organoheterocyclic compounds (12.9%). In total, 45 differential metabolites, representing 48.4% of all identified differential metabolites, were shared between the AS vs. C and PP vs. AS comparisons (Figure 5C). KEGG annotation showed that these metabolites were predominantly enriched in global and overview metabolic pathways (12 metabolites), lipid metabolism pathways (6 metabolites), and transport and catabolism pathways (6 metabolites; Figure 5D).

Among the shared metabolites, 13 were increased and 32 were decreased in the AS group compared with the C group, and these changes were reversed in the PP group (*p* < 0.05; Figure 6A). The upregulated shared metabolites in the AS group were dominated by glycerophospholipids (U7–U13, 7 types). Cytidine, ureidopropionic acid, propionylcarnitine, deoxycytidine, cytosine, and L‐Asp levels were also higher in the AS group than in the C group (U1–U6; Figure 6A). The relative contents of these metabolites were lower in the PP group than in the AS group (*p* < 0.05).

**FIGURE 6:**
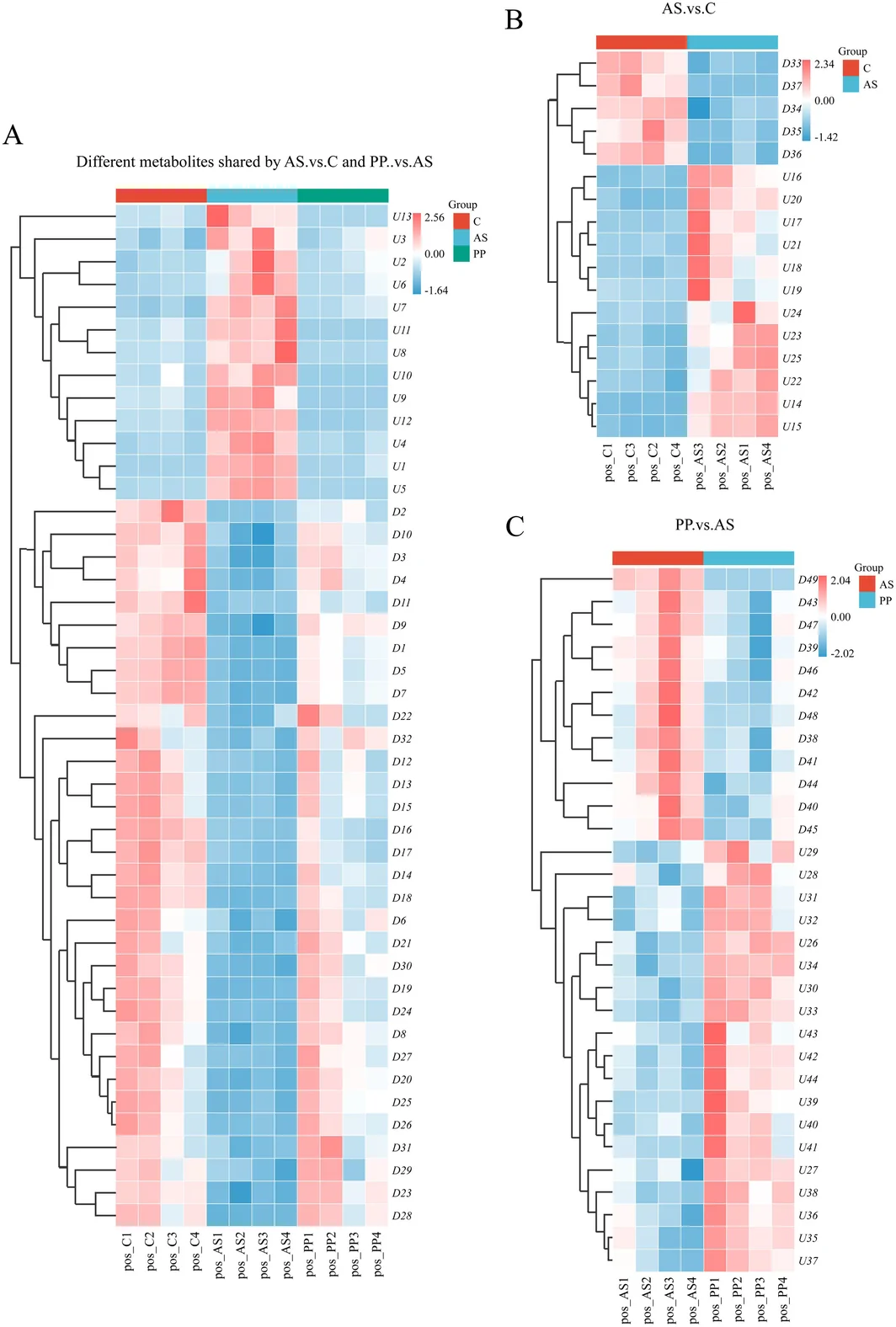
Hepatic metabolite changes among mice groups (*n* = 4 per group). (A) Heatmap of shared differential metabolites between PP and AS groups and AS and C groups. (B, C) Heatmaps of differential metabolites exclusively between AS and C groups (B) and PP and AS groups (C). Metabolite IDs U1–U44 and D1–D49 correspond to representative metabolites, including U1, cytidine; U2, ureidopropionic acid; U3, propionylcarnitine; U4, deoxycytidine; D1, indole‐3‐carboxaldehyde; D2, 4‐(2‐aminophenyl)‐2,4‐dioxobutanoic acid; D3, quinaldic acid; and D4, L‐kynurenine. The complete list of all 93 metabolites, including names, formulas, HMDB IDs, KEGG IDs, and Lipidmaps IDs, is provided in Table S2.

Conversely, 21 N‐acyl amino acids (D12–D32) were lower in the AS group than in the C group (Figure 6A). Indole‐3‐carboxaldehyde, 4‐(2‐aminophenyl)‐2,4‐dioxobutanoic acid, quinaldic acid, L‐kynurenine, indole‐3‐carboxylic acid, flavin adenine dinucleotide, indoleacrylic acid, nicotinamide adenine dinucleotide, 3‐methyluridine, 3‐methyldioxyindole, and glutathione were also lower in the AS group than in the C group (D1–D11; Figure 6A). The relative contents of these metabolites were higher in the PP group than in the AS group (*p* < 0.05).

In addition to the shared metabolites, 12 metabolites were increased and 5 were decreased in the AS group compared with the C group (*p* < 0.05). Glycerophospholipids and amino acids, peptides, and analogs were the primary metabolites and exhibited a notable increase in their relative contents (U18–U25; Figure 6B). D‐glucurono‐6,3‐lactone, 1‐(beta‐D‐ribofuranosyl)‐1,4‐dihydronicotinamide, acetylcarnitine, and allolithocholic acid also increased significantly (U14–U17; Figure 6B). In contrast, delta‐decalactone, quinoline‐4‐carboxylic acid, N‐acetyl‐L‐carnosine, arachidonoyl ethanolamide, and 3b‐hydroxy‐5‐cholenoic acid were decreased in the AS group compared with the C group (D33–D37; Figure 6B).

Beyond the shared metabolites, the relative contents of 19 metabolites increased and 12 decreased in the PP group compared with the AS group (*p* < 0.05). N‐Acyl amino acids were the most abundant class among the upregulated metabolites (U30–U44; Figure 6C). In addition, the relative contents of decanoylcarnitine, 1‐methyl‐2‐nonyl‐4(1H)‐quinolinone, castanospermine, and alpha‐linolenic acid also increased significantly (U26–U29; Figure 6C). Conversely, 11 amino acids, peptides, and analogs were decreased in the PP group compared with the AS group (D38–D48; Figure 6C), as was allopurinol (D49; Figure 6C).

### 3.6 Compositional Features of P‐MRPs

A total of 27 small‐molecule compounds with significant abundance changes were identified in P‐MRPs (Figure 7A; detailed information is provided in Table S1). These compounds were classified into several major categories, including small‐molecule peptides (10 compounds), amino acids and derivatives (7), indole derivatives (3), lipid degradation products (3), sugars and sugar derivatives (2), and alkaloids (2). Among the small‐molecule peptides and amino acid derivatives, the most pronounced abundance changes after the Maillard reaction were observed for N‐acetyl‐Cys (FC = 1929.52, ranked first among the 27 compounds), Arg (FC = 119.81, ranked second), Cystine (FC = 59.13), Leu‐Leu‐Tyr (FC = 57.68), N‐acetyl–Gly (FC = 27.78), N‐acetyl–Tyr (FC = 19.77), Glu‐Thr (FC = 11.72), and Asp‐Glu (FC = 11.36; Figure 7B).

**FIGURE 7:**
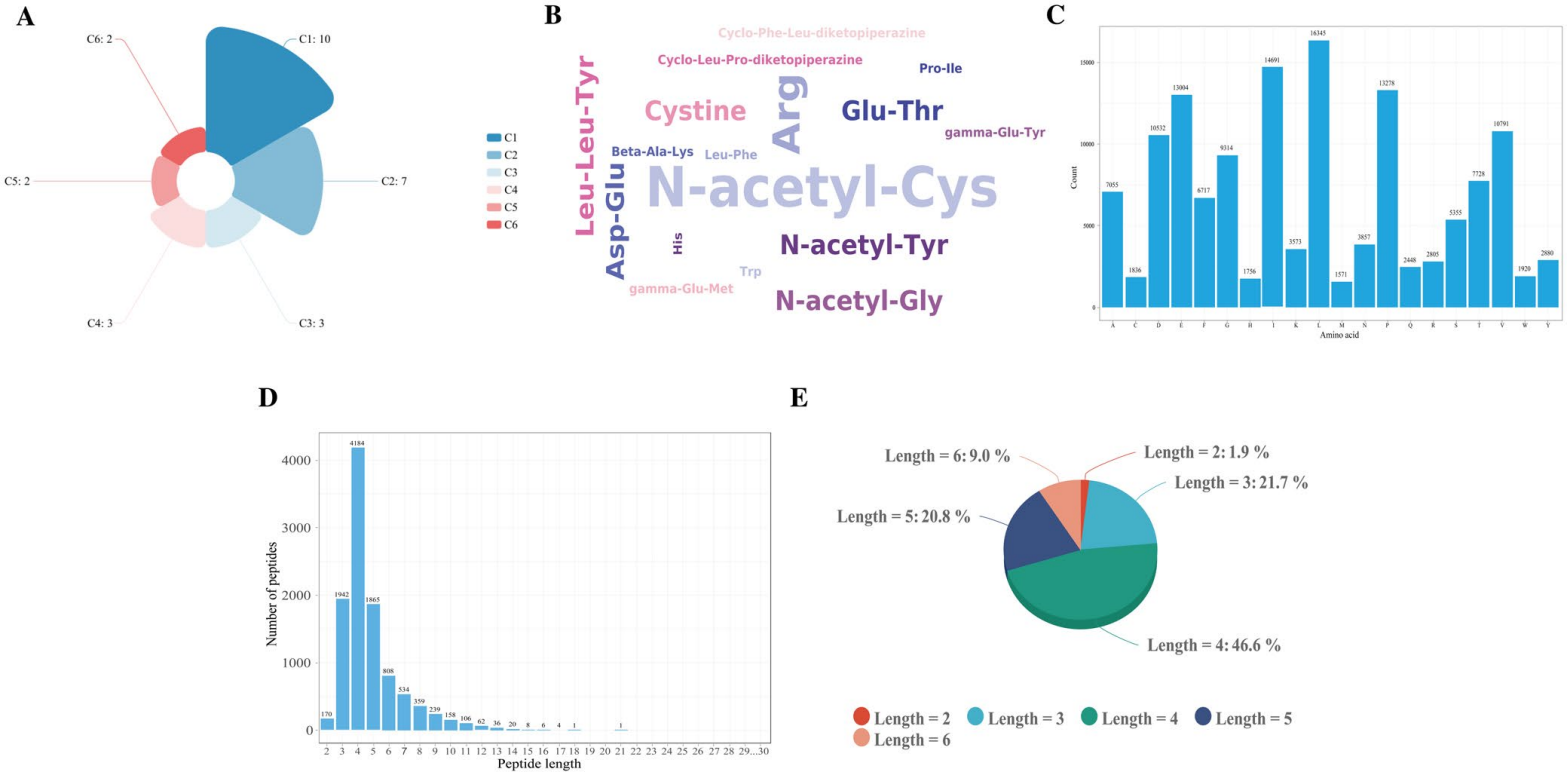
Compositional characteristics of P‐MRPs (*n* = 3 per group). (A) Classification of 27 small‐molecule compounds with significant abundance changes in P‐MRPs: C1, small‐molecular peptides; C2, amino acids and their derivatives; C3, indole derivatives; C4, lipid degradation products; C5, sugars and sugar derivatives; and C6, alkaloids. (B) Fold change values of small‐molecular peptides and amino acid derivatives after the Maillard reaction; larger font size indicates greater fold change. (C) Amino acid composition of identified peptides in P‐MRPs. (D) Length distribution of all identified peptides. (E) Length distribution of small‐molecular peptides (2–6 residues). P‐MRPs, *P. aibuhitensis*‐derived Maillard reaction products.

Peptidomic profiling revealed that essential amino acids accounted for 44.6% of the total amino acid content across all identified peptides, with branched‐chain amino acids (leucine, isoleucine, and valine) comprising 66.0% of this fraction (Figure 7C). Most peptides in P‐MRPs were small molecules containing 2–6 amino acid residues, representing approximately 85.4% of all identified peptides (Figure 7D). Within this small‐molecule peptide fraction, peptides containing 3–5 amino acids were predominant, accounting for approximately 89.1% (Figure 7E).

## 4 Discussion

### 4.1 Overview of the Hepatoprotective Effects of P‐MRPs in ALD Mice

In this study, P‐MRPs administration attenuated alcohol‐induced liver injury in mice, as evidenced by reduced serum ALT and AST activities, improved histopathology, restored antioxidant enzyme activities, and decreased pro‐inflammatory cytokine levels. These phenotypic improvements prompted further investigation of the metabolic pathways affected by P‐MRPs treatment. Metabolomic profiling identified 91 and 89 differential metabolites in the AS versus C and PP versus AS comparisons, respectively. The affected pathways were primarily associated with oxidative stress, lipid metabolism, and inflammatory regulation, which are central processes to ALD pathogenesis (Zhou et al. 2026; Wang et al. 2024; Zhu et al. 2026). To link the metabolomic findings with phenotypic outcomes, we assessed hepatic protein levels of key regulatory nodes in these pathways, including NRF2 for antioxidant defense, NF‐κB p65 and TNF‐α for inflammatory signaling, and CPT1A for lipid metabolism. Together, the integrated data suggest that P‐MRPs modulate multiple interconnected pathways to alleviate alcohol‐induced liver injury.

### 4.2 P‐MRPs and Hepatic Oxidative Stress

Oxidative stress is a key pathogenic mechanism in ALD, driven by ethanol‐induced ROS generation and impaired antioxidant defenses (Michalak et al. 2021; Han et al. 2020). In the present study, alcohol feeding significantly decreased hepatic NRF2 protein levels, accompanied by reduced SOD and GPx activities and elevated MDA levels. P‐MRPs administration restored NRF2 protein levels, increased SOD and GPx activities, and reduced MDA levels.

NRF2 is a master transcription factor that regulates the expression of antioxidant enzymes, including SOD and GPx (Di Meo et al. 2019). The concurrent recovery of NRF2 and downstream antioxidant enzymes in P‐MRPs–treated mice suggests that P‐MRPs may help preserve NRF2‐mediated antioxidant defense. The reduction in hepatic MDA—a biomarker of lipid peroxidation—further supports the attenuation of oxidative damage (Kudoh et al. 2014).

Metabolomic analysis revealed that P‐MRPs intervention restored key tryptophan‐derived metabolites reduced by alcohol exposure, including L‐kynurenine and indole‐3‐carboxaldehyde. Tryptophan metabolism contributes to redox regulation through the kynurenine and indole pathways (Arto et al. 2024; Tan et al. 2025). Indole‐3‐carboxaldehyde exerts protective effects against liver injury by reducing oxidative stress and hepatocyte apoptosis (Liu et al. 2024). L‐kynurenine and related metabolites have also been associated with antioxidant and immunoregulatory effects in liver disease (Tan et al. 2025). The recovery of these metabolites in the PP group is consistent with the observed reduction in oxidative stress markers.

The antioxidant effect of P‐MRPs was also validated in AML12 hepatocytes, where P‐MRPs treatment reduced ethanol‐induced ROS accumulation and attenuated ALT and AST leakage (Figure S2). These findings indicate that P‐MRPs exert direct cytoprotective effects on hepatocytes independent of systemic factors. Collectively, the concordance among NRF2 restoration, antioxidant enzyme recovery, metabolomic changes, and in vitro cytoprotection supports the involvement of NRF2‐mediated and tryptophan metabolism‐related pathways in the antioxidant effects of P‐MRPs.

### 4.3 P‐MRPs and Hepatic Inflammation

Chronic alcohol consumption promotes hepatic inflammation through activation of pro‐inflammatory signaling pathways (Patel et al. 2026). In the current study, P‐MRPs intervention reduced hepatic NF‐κB p65 protein levels, accompanied by decreased TNF‐α, IL‐6, IL‐1β, and IL‐18 levels, all of which are downstream effectors of NF‐κB signaling (Liu et al. 2021; Schmitz and Baeuerle 1991). NF‐κB is a central transcription factor that regulates pro‐inflammatory cytokine expression (Mitchell and Carmody 2018), and NF‐κB p65 is the core functional subunit responsible for transcriptional activation (Schmitz and Baeuerle 1991). The concurrent reduction in NF‐κB p65 protein levels and downstream cytokines suggests that P‐MRPs may suppress alcohol‐induced hepatic inflammation.

Metabolomic analysis showed that alcohol feeding depleted multiple N‐acyl amino acids, whereas P‐MRPs treatment restored their levels. N‐acyl amino acids are endogenous lipid mediators involved in inflammatory regulation (Arul Prakash and Kamlekar 2021; Battista et al. 2019; Burstein 2018). Several species, including N‐palmitoyl, N‐stearoyl, N‐oleoyl, N‐linoleoyl, N‐arachidonoyl, and N‐docosahexaenoyl amino acids, were significantly decreased in alcohol‐fed mice and restored in the PP group. The coordinated restoration of these lipid mediators was associated with reduced pro‐inflammatory cytokine production and attenuated histopathological inflammation. Recovery of indole metabolites, including indole‐3‐carboxaldehyde, indoleacrylic acid, and indole‐3‐carboxylic acid, further supports the anti‐inflammatory phenotype (Amarasiri et al. 2025; Luo et al. 2024; Puccetti et al. 2025).

Collectively, these observations suggest that P‐MRPs may alleviate alcohol‐induced hepatic inflammation by suppressing NF‐κB signaling and restoring two classes of endogenous anti‐inflammatory mediators: N‐acyl amino acids and indole metabolites. The convergence of these metabolite changes with reduced NF‐κB p65 protein levels and pro‐inflammatory cytokine production highlights the multi‐target nature of P‐MRPs intervention in attenuating alcohol‐associated hepatic inflammation.

### 4.4 P‐MRPs and Hepatic Lipid Metabolism

Hepatic lipid accumulation is a hallmark of ALD, partly resulting from increased lipogenesis and impaired fatty acid oxidation (Dunn et al. 2025). In the present study, alcohol exposure increased serum TC and TG levels, whereas P‐MRPs administration significantly reduced both parameters. Metabolomic analysis further showed that alcohol feeding increased several glycerophospholipid species, particularly lysophosphatidylcholines (LysoPCs) and lysophosphatidylethanolamines (LysoPEs), consistent with previous reports in alcoholic fatty liver models (Zhao et al. 2011). P‐MRPs intervention reversed these changes, suggesting attenuation of alcohol‐induced membrane phospholipid remodeling.

Alcohol exposure also increased acylcarnitines, including propionylcarnitine and acetylcarnitine. Given that acylcarnitine accumulation reflects incomplete fatty acid oxidation and mitochondrial dysfunction (Shi et al. 2021), the reduction of these metabolites in the PP group suggests improved mitochondrial β‐oxidation. Consistent with this interpretation, hepatic CPT1A protein levels, which were decreased by alcohol feeding, were restored by P‐MRPs administration. As CPT1A is the rate‐limiting enzyme for mitochondrial fatty acid uptake and is commonly downregulated in alcoholic fatty liver (Hu et al. 2013; McGarry and Brown 1997), its recovery supports a role for P‐MRPs intervention in improving mitochondrial fatty acid oxidation capacity.

Overall, these findings indicate that P‐MRPs regulate lipid metabolism in ALD, potentially by restoring CPT1A‐mediated fatty acid oxidation and reducing glycerophospholipid remodeling. Together with decreased oxidative stress and inflammation, these lipid metabolic improvements may contribute to attenuating alcohol‐induced hepatic injury.

### 4.5 Compositional Features of P‐MRPs as a Resource for Future Investigation

Metabolomic and peptidomic profiling identified 27 small‐molecule compounds with significant abundance changes in P‐MRPs, including small‐molecule peptides, amino acid derivatives, and indole derivatives. N‐acetyl–Cys, Arg, cystine, Leu‐Leu‐Tyr, N‐acetyl–Gly, N‐acetyl–Tyr, Glu‐Thr, and Asp‐Glu showed the most pronounced abundance changes after the Maillard reaction. Peptidomic analysis further showed that P‐MRPs were enriched in essential amino acids (44.6%) and branched‐chain amino acids (66.0% of essential amino acids), with small‐molecule peptides containing 2–6 residues accounting for 85.4% of all identified peptides. Several identified compounds have reported hepatoprotective or anti‐inflammatory activities in liver injury models. For example, N‐acetyl–Cys attenuates alcohol‐induced hepatic lipid accumulation and oxidative damage (Wang et al. 2023), whereas arginine supplementation ameliorates ethanol‐induced hepatic inflammation and fibrosis (Daff et al. 2026; Nanji et al. 2001). Marine‐derived small‐molecule peptides can modulate processes related to liver injury (Chen et al. 2025; Siregar et al. 2021; Xiao et al. 2023). However, the contributions of individual components to the hepatoprotective effects observed in this ALD mouse model remain unclear. These compositional profiles provide candidate compounds for future activity‐guided fractionation and bioassay‐directed validation.

### 4.6 Limitations and Future Directions

Certain limitations of this study should be considered. First, metabolite annotations were putative at MSI Level 2 and should be confirmed using authentic standards, particularly for isomeric compounds. Second, the proposed antioxidant, anti‐inflammatory, and lipid‐regulatory mechanisms are based on metabolomic and biochemical associations rather than direct causal evidence. Although protein‐level measurements of NRF2, NF‐κB p65, TNF‐α, and CPT1A provide correlative molecular evidence, targeted studies are required for definitive mechanistic validation. Third, the metabolomics sample size (*n* = 4 per group), although supported by PCA separation and QC consistency, should be expanded to improve statistical power. Fourth, the absence of in vivo non‐Maillard hydrolysate (PEH‐HDH) and amino acid/sugar control groups limits attribution of the observed effects specifically to Maillard reaction products, although P‐MRPs showed stronger protective effects than PEH‐HDH in AML12 hepatocytes. Fifth, the bioavailability and metabolic fate of P‐MRPs and their components remain unknown. Finally, systematic toxicity and dose–response studies are needed.

Despite these limitations, this study provides an integrated characterization of P‐MRPs–mediated hepatoprotection in a mouse model of ALD by combining phenotypic, biochemical, protein‐level, metabolomic, and compositional data. These findings generate testable hypotheses regarding NRF2‐mediated antioxidant responses, NF‐κB–driven inflammatory pathways, and CPT1A‐regulated lipid metabolism that warrant further mechanistic and translational investigation.

## 5 Conclusion

This study demonstrates that P‐MRPs protect against alcohol‐induced liver injury in mice, as indicated by lower serum ALT and AST activities, improved hepatic histopathology, restored antioxidant enzyme activities, and reduced pro‐inflammatory cytokine levels. Integrated metabolomic and protein‐level analyses suggest that P‐MRPs intervention targets three interconnected processes central to ALD progression: oxidative stress, by restoring NRF2 signaling and tryptophan‐derived metabolites; hepatic inflammation, by suppressing NF‐κB p65 and restoring N‐acyl amino acids and indole metabolites; and lipid metabolic dysfunction, by improving CPT1A‐mediated fatty acid oxidation. In vitro experiments further demonstrated direct antioxidant and cytoprotective effects of P‐MRPs in hepatocytes. Compositional profiling identified 27 small‐molecule compounds with significant abundance changes in P‐MRPs and showed enrichment of small‐molecule peptides, providing a preliminary basis for future activity‐guided investigations. Although these findings remain correlative and require mechanistic validation, they provide a hypothesis‐generating framework for future investigation of P‐MRPs‐mediated hepatoprotection.

## Author Contributions

**Tonglei Qiao:** conceptualization, methodology, software, data curation, formal analysis, project administration, resources, validation, visualization, investigation, funding acquisition, writing – original draft, writing – review and editing. **Yuchun Lu:** conceptualization, investigation, methodology, resources, software, validation, visualization, formal analysis, funding acquisition, writing – review and editing. **Teng Teng:** conceptualization, methodology, formal analysis, investigation, resources, validation, software, visualization, writing – review and editing. **Zichen Deng:** conceptualization, formal analysis, investigation, methodology, software, validation, writing – review and editing. **Feng Liu:** conceptualization, supervision, writing – review and editing, project administration, resources, funding acquisition. **Chun‐E Liu:** resources, project administration, methodology, supervision, funding acquisition, writing – review and editing.

## Funding

This work was supported by the Natural Science Foundation of Shandong Province, China (grant number: ZR2021MC131), Shandong Province Leading Enterprise Project in Aquatic Seedlings Industry (Grant number: Lunong 202351‐21), Yantai “Double Hundred Plan” Blue Industry Leading Talent Project (Grant number: Yan Fa Gai 202518‐01), Yantai Aquatic Seedlings Industry Enhancement Project (Grant number: Yan Hai Yu 20241230), and Yantai City School‐Local Integration Development Project (Grant number: 2024XDRHXMP11).

## Ethics Statement

All animal experiments were conducted in compliance with the guidelines of the Animal Ethics Committee of China Agricultural University and Suzhou Weiyuan Biotechnology Co. Ltd., the ARRIVE guidelines, and the National Research Committee guide for the care and use of experimental animals (approval number: IACUC‐20240929‐001).

## Conflicts of Interest

The authors declare no conflicts of interest.

## Supporting information


**Table S1:** Significantly different substances between P‐MRPs and PEH‐HDH.
**Table S2:** Detailed information of differential metabolites between groups.
**Figure S1:** Pearson correlation analysis of quality control (QC) samples in positive ion mode. Pairwise Pearson correlation coefficients were calculated based on the relative quantitative values of all detected metabolites among QC samples. Each cell in the heatmap represents the correlation coefficient (*r*) between two QC injections. Darker color indicates a higher correlation.
**Figure S2:** Effects of different test substances on AML12 mouse hepatocytes. (A) Cell viability following ethanol treatment at 0, 31.25, 62.5, 125, 250, and 500 mmol/L. (B) Cell viability following PEH‐HDH treatment at 0, 50, 100, 200, 400, and 800 mg/mL. (C) Cell viability following P‐MRPs treatment at 0, 50, 100, 200, 400, and 800 mg/mL. (D) ROS levels in cell supernatants. (E) ALT activity in cell supernatants. (F) AST activity in cell supernatants. Ethanol at concentrations ≥ 250 mmol/L significantly reduced cell viability (*p* < 0.05; A), while neither PEH‐HDH nor P‐MRPs exhibited significant cytotoxicity at concentrations below 100 mg/mL (*p* ≥ 0.05; B, C). Based on these results, 250 mmol/L ethanol and 100 mg/mL PEH‐HDH/P‐MRPs were selected for subsequent experiments. Compared with the alcohol‐treated group, P‐MRPs co‐incubation significantly reduced ROS levels and attenuated ALT and AST elevation (D–F; *p* < 0.05), with P‐MRPs showing greater protective effects than PEH‐HDH. Data are presented as mean ± SEM (*n* = 6 per group). Different lowercase letters above bars indicate significant differences (*p* < 0.05, one‐way ANOVA with Tukey's test).

## Data Availability

The data that support the findings of this study are available from the corresponding author upon reasonable request.
