# Supplementary material for: Hepatoprotective Effects of Maillard Reaction Products From Perinereis aibuhitensis: Identification and Metabolomic Insights in a Mouse Model of Alcoholic Liver Disease
Source: Food Sci Nutr. 2026 Oct 7;14(10):e72417. doi: 10.1002/fsn3.72417 (PMC13646695; doi:10.1002/fsn3.72417)
Supplement: Supplementary file 1 [file FSN3-14-e72417-s001.docx]

**S1. Supplementary tables**

**Table S1.** Significantly different substances between P-MRPs and PEH-HDH

| **Metabolite name** | **Average Rt(min)** | **Average Mz** | **HMDB** | **MS1 isotopic spectrum** | **MS/MS spectrum** | **FC** |
| --- | --- | --- | --- | --- | --- | --- |
| N-acetyl-Cys | 1.419 | 162.02179 | HMDB0001890 | 162.02179:15511859 163.02514:988818 164.0285:874130 | 55.01744:38933 57.03297:315556 58.03653:16149 59.01221:864088 60.01585:35508 67.01757:29651 68.99632:38796 69.03284:71595 71.01242:507423 71.04877:8361 72.01562:32467 72.99145:31497 73.02811:1113884 74.03144:80875 75.00687:93582 81.03288:39826 83.01224:99475 84.04384:441093 85.02787:850594 85.04719:16934 86.03149:66548 87.00757:47906 87.04381:11064 89.02312:118836 90.02641:8452 91.03854:10648 91.05418:27191 95.01273:35264 97.02837:51490 99.00771:11773 99.04435:30911 101.02329:545757 102.02637:26611 103.0382:35983 105.01782:34835 113.02306:740616 114.02671:87655 115.02747:11815 115.03917:12007 118.03949:23160 119.03452:14925 119.04929:16851 120.01099:9648 131.03389:142565 132.03667:22759 143.03284:11082 161.04391:75641 162.02159:38185 162.04068:14652 162.06612:12828 | 1929.521479 |
| Arg | 0.839 | 175.11899 | HMDB0000517 | 175.11899:138616768 176.12234:15238139 177.1257:695606 | 56.05038:16370 58.06606:15015 60.04481:116680 60.05628:5426458 61.05344:31515 61.05967:38835 67.05513:17155 68.05034:17583 68.98302:23720 70.0506:100434 70.06609:9180469 71.04984:128000 71.06284:22567 71.06908:292981 72.08158:523526 73.08504:18179 74.02435:23035 74.0606:10894 84.08153:813003 84.96074:16003 85.06556:16461 85.08482:36313 86.09727:63680 86.99282:70146 88.07654:51720 95.06088:11431 97.07646:65614 98.06087:53156 98.96027:19447 100.02477:11197 100.07612:35559 105.00316:77169 106.00416:13669 110.07174:25161 112.08693:309531 113.07135:32036 113.09077:12983 114.10252:76997 115.08661:83660 116.03867:21325 116.07069:2404035 117.07433:78473 119.01958:10620 128.01888:42686 129.02112:15365 129.10178:27931 130.08681:199120 130.09807:680983 130.12157:36365 131.10025:29054 132.0507:31497 133.09741:10906 141.06497:12925 143.10335:8984 147.01338:15903 157.08174:31455 157.10788:96816 158.09297:508940 159.09506:24727 160.04521:20775 161.02907:15045 161.11244:13852 162.0416:11567 165.02457:15296 175.11819:2635332 175.14456:135171 | 119.8108815 |
| Cystine | 0.821 | 239.01625 | HMDB0000192 | 239.01625:78883624 240.0196:11039929 241.02296:19279626 | 50.15289:34404 52.09549:29781 56.3188:29730 61.48674:33933 64.95116:969382 71.14145:33318 71.99001:375328 74.0055:6116572 75.00877:52635 75.90887:37449 78.95751:1154870 86.02322:915808 86.15425:37097 93.69425:34140 96.96851:56564 97.36546:35831 117.9958:167463 120.01102:4035829 121.0147:43929 140.974:32974 151.98325:391563 157.703:35705 158.32085:33340 166.80066:37526 171.00531:146071 193.21799:35952 217.76599:32730 238.9048:112200 239.01704:616566 | 59.12517254 |
| Leu-Leu-Tyr | 6.328 | 408.24832 | HMDB0304804 | 408.24832:75176992 409.25167:20127288 410.25503:2845554 | 51.39447:17692 56.05046:40755 69.07069:56554 70.06565:78053 70.34796:16849 72.08167:114924 84.0816:127892 86.0974:562903 123.02306:22380 129.10196:23580 167.73795:23311 229.84897:19263 398.59998:21599 | 57.68400113 |
| N-acetyl-Gly | 0.975 | 233.0775 | HMDB0000532 | 233.0775:18495604 234.08085:0 235.08421:0 | 51.96487:3949 57.03296:8660 58.02816:17362 59.01204:20122 64.45215:4379 66.03326:6652 68.01295:6097 68.04913:14473 69.03281:6460 70.02853:16013 71.01245:17950 71.0238:7746 72.00735:31805 72.99146:5766 73.03927:84627 74.02291:230725 74.98967:15590 75.02663:8444 76.02754:3848 78.91756:8408 78.95747:11379 79.95623:8308 80.04852:4675 80.91537:16956 80.96371:8330 82.02827:31289 83.0237:5449 84.0079:23648 84.04375:48264 85.02785:8794 85.0389:36310 86.0232:9905 86.05952:7429 87.05526:103263 88.03883:38414 89.0231:9665 94.02798:5490 96.00768:10296 96.04427:7157 96.96851:16942 97.03918:6335 98.02287:21377 98.05977:8058 99.05536:32531 100.0207:9835 100.03873:9013 101.07041:24879 102.05457:149805 102.07331:6508 103.04944:9896 104.03349:194856 105.03704:6120 108.04427:32168 110.02338:5924 110.09658:31557 111.05498:8734 111.09938:8639 112.03927:15282 112.93648:4767 113.03436:35026 113.0709:15373 115.05089:4772 115.0858:45974 117.06532:8382 118.04923:13049 124.0396:6137 125.07048:16690 126.05492:79071 127.05022:11642 127.0869:18087 128.03355:90731 129.06622:5629 130.08594:4866 131.04518:6684 138.17346:4012 141.06592:44889 146.04512:21899 153.06557:13799 154.05086:7140 154.08647:35916 155.11896:15576 156.065:6688 158.0925:6273 159.07729:28051 170.05707:5134 171.04137:13919 171.0766:24575 173.05736:16529 185.0549:63514 187.07228:6341 187.10869:7423 188.0657:24166 188.1129:18798 189.08742:24475 189.11252:4416 197.05661:7169 199.07356:10816 202.08261:18241 203.06616:27008 215.06651:81345 216.07234:4618 232.09543:47264 232.13586:8590 233.07788:267108 233.15552:11111 | 27.78481261 |
| N-acetyl-Tyr | 6.886 | 445.15567 | HMDB0000866 | 445.15567:17408840 446.15902:4655248 447.16238:406222 | 58.02816:4183 61.98679:11392 64.42664:4404 66.03326:10630 67.02853:4874 70.02853:14041 72.0074:17724 73.03939:13580 74.02309:84195 79.95624:12583 82.0284:88064 84.04385:10265 86.0232:6523 88.03883:7300 91.0542:4330 94.02799:8326 96.04427:5662 96.95856:14918 99.01894:4829 99.05575:22752 103.05044:6552 106.06543:9327 109.0396:27415 110.0234:25120 111.01869:25772 111.05498:19155 112.03924:20123 114.05458:75190 116.04986:87695 120.01101:13759 124.0396:6045 125.03468:7728 127.05031:73609 129.10239:9323 130.08618:34283 131.08931:6448 139.08675:10378 141.0661:14214 142.06445:17351 149.07236:4795 150.05571:19922 157.07571:5573 159.09254:11979 165.05347:4706 167.0809:7283 183.0779:6367 186.12231:5011 192.07701:25051 193.06271:10090 197.07761:5364 198.05992:12150 199.18283:10365 200.18362:11759 203.08371:132004 204.08615:7375 206.09157:10260 210.08885:32523 217.13348:12499 224.10596:9233 238.15715:6461 240.08205:52768 241.06616:279008 242.06653:10034 242.13083:4634 246.14986:5401 254.09407:15074 254.18628:6222 258.09317:13751 261.12378:6567 262.12305:6075 268.11172:8660 269.11792:5149 270.12558:15773 272.10812:21936 284.11047:5151 296.10797:7485 313.26822:6991 316.10062:32955 321.19751:4997 353.16446:15420 365.22162:6565 366.22318:13829 377.18384:5894 401.16693:55624 402.16708:6847 411.17255:6771 414.28165:12408 444.198:6271 444.29703:25824 445.15833:305186 | 19.77459679 |
| Glu-Thr | 0.991 | 247.09317 | HMDB0028829 | 247.09317:36226528 248.09652:5074073 249.09988:0 | 56.04902:19214 58.02816:5986 58.99459:3754 59.01221:9458 66.03326:4475 68.04882:39469 70.02798:7585 71.01188:9777 72.0074:10620 73.0388:7316 74.02326:337281 74.02966:4964 74.98905:3821 78.95774:24644 79.95555:14192 80.96371:4031 82.02801:27967 82.06461:64027 84.00732:13187 84.04417:20989 85.02711:9091 85.03902:13408 86.02245:3923 87.05471:20499 88.03883:11498 89.02231:4978 91.0369:3737 94.02798:4545 95.0593:4192 96.95855:10964 96.96805:24606 98.00546:9088 98.02344:23586 98.05977:5145 100.03873:7990 101.02233:4161 102.05416:33346 103.05738:3383 107.97411:4598 112.03927:6680 113.03329:9200 113.0703:25157 115.08601:9700 116.06992:9610 117.07372:7349 118.04943:16307 119.5969:3053 123.0434:3623 123.05376:8842 124.0396:3456 125.07108:74833 126.05448:25443 128.03438:56637 131.04376:3163 139.08603:14253 141.06531:18650 141.10106:4607 146.02669:6117 146.04434:14136 157.06137:44224 159.07352:3736 167.0338:7422 169.06134:146423 170.08022:8020 173.05519:5989 185.05629:45474 185.09116:3627 186.05971:3265 199.1082:7794 201.02431:3472 201.08649:11181 203.04933:70235 204.03513:15290 213.08722:40249 228.05157:4737 228.10712:8469 229.08057:5062 229.98088:4879 230.04704:5185 246.04367:11699 246.1169:12231 247.02382:19687 247.07521:152515 247.11589:16443 | 11.7204337 |
| Asp-Glu | 1.012 | 261.07321 | HMDB0028752 | 261.07321:27819926 262.07656:3966022 263.07992:4708447 | 55.11786:5040 58.02816:9375 59.01221:21967 63.94333:32517 64.95104:34799 68.04913:10744 70.02853:10294 71.01243:146736 71.98944:10537 73.03937:53310 74.0055:8300 74.02293:37810 74.98973:76337 78.91766:27111 78.95747:124096 79.93793:17679 79.95624:16326 80.91537:8037 82.02837:15281 84.04385:18338 85.02786:11628 86.0058:6190 86.0232:8020 87.05484:34941 88.03886:309428 89.02311:14713 94.02798:6759 96.00768:10019 96.04427:11291 96.9585:40765 96.96861:68778 97.03918:22131 98.02294:14065 99.05458:77969 100.02165:15050 101.07056:38579 102.05473:24482 103.05837:10198 104.20287:5925 111.05495:78844 112.03927:19633 113.03478:34610 114.01865:7578 115.00181:93445 115.04987:79503 119.04931:5576 127.05022:11938 128.03394:36128 129.03703:6269 129.10239:8245 130.08594:23597 131.08931:20220 132.02948:28529 138.05341:7318 139.08675:8682 145.04295:29827 145.0609:21009 146.02669:10318 146.04509:13441 171.07718:29492 173.05519:6885 173.96553:20535 183.07587:35024 187.0723:24430 196.79979:5815 198.13132:6344 202.01724:5948 216.14165:17092 217.06371:5704 226.08521:8675 227.06833:65855 231.06294:9540 232.02858:5739 242.12434:30124 243.04095:8528 243.12361:8568 245.29144:4958 260.02283:14850 260.07849:18782 260.13434:80825 260.99976:15686 261.04376:34382 261.05576:204935 261.13116:27706 | 11.35857006 |
| Cyclo-Leu-Pro-diketopiperazine | 6.651 | 211.1442 | HMDB0034276 | 211.1442:78771424 212.14755:11993439 213.15091:19953356 | 57.03432:6575 67.05521:17683 68.05042:6493 69.03477:8638 69.07064:39126 70.06618:957498 71.06918:22276 72.04508:38190 79.05475:8891 81.03445:10240 83.04925:8762 84.08165:12050 86.09736:389728 87.10012:8510 91.05462:8099 95.0487:6653 98.06097:100645 102.01818:9059 105.07062:9195 109.43285:6563 114.092:39936 127.08628:8845 138.12775:40498 154.07291:14732 155.15416:12577 183.14946:70179 194.1176:13415 211.1447:662678 | 3.507680493 |
| gamma-Glu-Tyr | 5.368 | 309.10913 | HMDB0011741 | 309.10913:6591724 310.11248:945264 311.11584:0 | 58.02816:3298 61.98679:5744 64.64101:3141 70.02853:5487 72.00741:7406 73.02815:3627 73.03938:91560 74.02302:9961 78.36073:2908 80.26563:2790 87.05461:8079 93.03294:4846 93.40337:3110 96.95856:3405 97.03919:11449 98.02295:6028 108.79486:3565 115.04978:46141 119.04955:18030 128.03374:4496 130.06062:8813 133.67331:3308 163.03902:5917 166.42133:2944 180.06616:32402 193.09856:29683 225.0347:3708 225.40973:2742 234.12416:4666 235.10817:17888 242.04869:7381 272.1622:5516 278.11688:3486 279.10037:82171 280.10242:5260 308.12735:16491 309.10638:33955 | 3.368898406 |
| His | 0.819 | 156.07663 | HMDB0000177 | 156.07663:51261404 157.07998:6316564 158.08334:4316502 | 54.03491:36945 56.05039:272980 67.05517:29912 68.05038:288203 70.06615:31908 72.08163:25397 81.04546:168696 82.05331:776747 83.06142:2570273 84.06461:82013 84.08159:29996 85.07677:28843 93.04544:1490802 94.04853:52267 95.06097:2227211 96.06387:57909 110.04005:170816 110.07185:21660424 111.05569:86383 111.07195:190590 111.07528:760183 112.08697:27674 129.08794:26406 138.05235:189915 139.04976:20803 155.07928:103221 156.07658:1840921 | 3.169233638 |
| Pro-Ile | 1.319 | 229.15416 | HMDB0304810 | 229.15416:65582920 230.15751:7911986 231.16087:2128474 | 55.05539:20546 56.05045:15853 57.03432:13645 58.06613:11907 60.12526:9599 69.07073:66878 70.06619:2159514 71.06931:57430 72.08167:54256 81.03445:25143 84.0451:34980 84.08165:28258 84.46981:11189 86.09733:396186 87.10012:14427 87.25993:10857 92.41116:12084 99.04448:12748 116.07076:135067 132.0408:14688 168.82906:12367 211.7455:13614 215.81261:12193 221.16937:12202 229.15318:28114 | 3.062173737 |
| Beta-Ala-Lys | 0.793 | 216.13515 | HMDB0060442 | 216.13515:20008058 217.1385:9673867 218.14186:701115 | 54.15885:3432 59.01221:4769 61.98678:78006 70.02852:14214 71.01245:4467 72.99144:192882 73.99946:3916 75.84931:3049 84.64031:2939 87.0546:72082 88.03891:59891 89.02306:144525 97.07545:10932 99.09186:5932 99.61308:2865 102.98697:6130 104.95848:6017 111.98755:3199 120.95412:8938 122.68179:3580 129.99873:3346 131.08076:4077 139.81293:3034 144.11287:3119 145.09727:66064 148.94815:4317 155.11713:11078 156.11308:4355 157.99271:14787 160.97789:3930 172.1454:102796 173.14807:7644 187.34921:3215 188.9738:15517 200.04134:6040 215.12965:9432 216.1356:608206 | 3.034237896 |
| Leu-Phe | 6.275 | 279.16956 | HMDB0302841 | 279.16956:232062368 280.17291:44442392 281.17627:4424277 | 56.05044:92736 57.058:70704 57.07072:548350 58.06611:66809 67.05467:45047 69.07068:2348519 70.06616:395344 70.07397:54154 72.08165:101003 73.65013:33190 84.08163:132634 85.02919:142800 86.07385:206270 86.09733:28159178 87.0947:68561 87.10005:712166 92.41787:41523 100.02202:81144 103.05408:51104 110.07185:81700 116.07081:56924 120.08087:1343511 121.02872:50742 121.0843:67018 124.23283:36158 129.1019:43951 139.9342:32387 149.02371:1526663 150.02612:63544 166.08536:698929 205.08609:34281 233.1662:117049 279.17154:1001900 | 2.926295493 |
| Trp | 2.814 | 243.05258 | HMDB0000929 | 243.05258:9389140 244.05593:953370 245.05929:786275 | 55.01899:3483 55.05501:7098 56.05046:10802 57.03432:5048 57.07075:17959 63.69566:2609 67.05521:7571 69.07056:14947 70.06565:8689 71.08624:5264 72.08168:3729 79.05542:2826 81.07044:4545 84.0452:138576 84.08178:19255 86.06026:3344 86.09743:149707 95.08562:4267 103.619:3033 121.01235:3192 126.40557:3272 132.10141:26132 164.08981:3008 197.12764:24985 242.10016:3456 243.13243:5202 243.23314:4462 | 2.620222415 |
| Cyclo-Phe-Leu-diketopiperazine | 0.751 | 261.15564 | HMDB0094673 | 261.15564:50595788 262.15899:9391932 263.16235:2973494 | 52.40972:3524 54.12888:3998 56.05043:25792 60.04531:27879 67.05521:6214 70.0295:10451 70.06636:22305 70.1564:3760 84.04511:41124 84.08167:480174 85.06567:4318 85.08508:21712 87.05637:28426 88.03989:39127 92.41116:4323 98.30634:4093 101.10763:14680 102.05533:15173 102.58344:4158 111.09204:6440 115.25446:4085 115.9855:4001 129.10199:86076 130.08557:12635 144.30931:4404 147.11337:9607 160.81438:4554 169.34621:4591 173.03601:4102 209.09384:4869 218.98332:9682 226.12061:6808 244.13145:6275 261.15494:40208 | 2.233990716 |
| gamma-Glu-Met | 1.046 | 277.08618 | HMDB0034367 | 277.08618:4568734 278.08953:0 279.09289:586449 | 54.57742:2724 57.09878:2931 59.42623:2941 61.55447:2600 61.98679:4432 73.0388:4751 74.02301:10289 78.95756:18242 79.95556:5707 82.02837:3601 85.75372:3049 87.54891:2938 89.02316:49053 96.95856:5774 96.96852:13546 114.05445:5186 115.20435:3307 127.05022:3205 128.03391:16153 141.84952:3528 146.22771:3038 148.04207:3825 148.39288:3177 164.0705:5031 215.08478:3827 219.0793:4208 241.06601:6401 259.07507:12764 276.08713:4745 277.08609:12453 277.12109:18966 | 2.156669043 |
| D-Glucose-6-phosphate | 6.488 | 259.02203 | HMDB0001401 | 259.02203:40711108 260.02538:4385660 261.02874:3239844 | 53.70512:6838 57.97412:21181 58.99459:16813 72.99147:9313 73.02815:9405 79.95556:15136 81.02039:16952 81.04453:67342 82.02838:126868 87.01303:27737 88.02076:78582 91.24732:7911 95.06027:101418 96.99751:19707 97.03919:7652 98.00546:10014 100.02071:19389 109.03956:9507 110.00587:10795 112.02134:280561 117.97764:38022 121.07659:11525 123.05505:281267 124.05926:10072 139.009:9979 149.99141:6779 153.04808:49770 156.05206:7072 169.04361:115800 171.88028:7357 173.84592:6759 181.04416:695625 182.04628:28071 191.77432:6948 198.27687:7158 213.03265:52942 215.03105:38866 225.03468:18707 244.3723:7160 259.02362:408011 259.11859:17579 | 104.0962543 |
| D-Mannitol | 7.499 | 227.07675 | HMDB0000765 | 227.07675:55034028 228.0801:5053141 229.08346:0 | 56.18197:225221 56.99663:390923 58.00427:434050 59.01221:354602 60.99137:253720 65.72192:240270 67.25549:237675 72.99147:26999690 73.02815:872647 73.99947:807758 75.00755:11192760 88.60085:214944 89.02312:8131516 94.1769:212911 95.92923:234715 104.66135:231731 110.09456:205161 177.09937:215892 181.07187:343563 181.12271:268969 200.38792:229479 227.13007:39637396 | 110.7139239 |
| Theophylline | 0.86 | 203.05215 | HMDB0001889 | 203.05215:498429280 204.0555:47647524 205.05886:3311825152 | 53.78792:59282 60.04531:157504 70.06572:2314506 71.06123:143628 72.08167:83773 78.36202:59894 81.03376:140800 84.04436:74021 84.08157:283068 85.02922:147608 86.0975:453440 88.08775:281904 93.96129:59309 112.11293:96393 116.07114:493118 139.23198:60664 144.10237:99351 145.88237:53237 197.9677:65533 202.10777:71137 203.05125:8861761 203.10338:219109 203.15042:544429 | 80.28526182 |
| Theobromine | 0.857 | 219.02609 | HMDB0002825 | 219.02609:96277696 220.02944:12221584 221.0328:443622400 | 53.25024:21329 56.855:18908 65.82159:20443 77.28573:18399 84.08166:31243 101.99376:27721 108.61556:21173 191.73367:22069 212.82208:20840 | 93.45715993 |
| Norharman | 0.679 | 186.10532 | HMDB0012897 | 186.10532:5196934 187.10867:1190098 188.11203:303129 | 55.01859:7111 55.05501:38211 56.05045:26114 57.0343:7884 57.07065:22787 58.06612:4060 59.04994:11710 60.0325:6545 60.04527:566621 60.05677:12573 61.04838:11291 67.05515:98573 68.05041:4650 68.9831:7087 69.0342:5403 69.07063:44488 70.02949:13208 70.06612:164182 71.0498:20920 71.06917:4880 71.08622:5685 72.0451:5515 72.08166:9562 79.0554:7074 81.03443:5719 81.07038:23959 82.05334:8840 82.06603:7590 83.04994:10521 83.06142:19503 83.08647:23707 84.04503:15501 84.08158:761248 84.96004:25933 85.0292:4616 85.06551:23243 85.08492:29618 86.0972:23712 86.99303:5594 88.03973:11573 91.0546:6550 93.04544:13499 93.07014:11730 95.04955:7419 95.06097:12542 95.08633:13563 97.0293:4729 97.06557:3899 97.10187:9647 98.06088:5652 99.04445:3931 101.06037:5048 101.1076:9892 102.09149:3744 105.0032:9461 106.04982:6269 107.08588:5774 109.10124:53092 109.95913:7431 110.07182:318599 111.04427:5692 111.07443:19152 112.07576:6699 112.08701:7448 113.06009:4176 116.07081:6075 116.96666:3624 122.07219:4900 126.0558:6785 126.0746:4163 128.10738:6290 129.10185:53574 130.08696:34316 138.06602:6921 140.06932:5198 142.59019:12781 144.99734:7761 145.13322:8347 147.11317:18524 153.25089:3480 156.07652:55115 166.06104:3943 167.09279:4695 172.9928:4284 173.11803:3744 175.11836:3879 176.27132:3396 179.09258:3936 185.1308:4919 186.07568:7121 186.09253:6873 186.11179:10371 186.14792:8405 | 5.53304385 |
| 3-Indolepropionic acid | 6.341 | 188.07445 | HMDB0002302 | 188.07445:18418154 189.0778:1429253 190.08116:727056 | 59.01221:5338 63.96085:3356 72.64909:2454 87.00764:2642 91.05338:4102 92.43336:5191 97.06458:4939 101.0416:9271 106.41332:2420 116.04859:9942 117.06893:3935 125.09573:19270 144.08008:7655 163.86343:2684 183.5864:2560 187.0972:14922 188.07466:17660 | 106.6835048 |
| (2-oxo-2,3-dihydro-1H-indol-3-yl)acetic acid | 1.24 | 192.06851 | HMDB0035514 | 192.06851:36727296 193.07186:2760822 194.07522:5154482 | 55.01896:15488 55.05536:21573 56.05042:28660 57.03428:21175 57.98812:20415 58.06604:100230 58.99574:1373600 59.04992:37736 59.9994:19877 60.99185:20418 61.01128:30564 67.05517:31485 68.05036:50558 69.03418:17218 69.07064:26501 70.06613:1202072 71.04984:252041 71.06914:27843 72.04507:25320 72.08163:15692 74.00686:151312 74.06065:27166 75.01456:74344 75.02704:41822 75.04478:24936 76.02233:110711 79.0547:30119 81.0344:22800 82.06587:81550 83.04991:17672 84.0816:26974 84.96005:25059 85.01124:50128 85.02917:17783 86.00646:239945 86.06099:39476 86.09724:75224 86.99066:915705 87.0268:55109 87.04459:16158 87.06844:261290 87.10084:21436 87.99427:14356 88.02246:14015 88.03975:145768 88.07652:1698812 89.07977:46649 91.0546:57123 94.06576:72085 95.04951:22839 95.07331:101368 98.06084:22150 99.04441:21306 100.02193:102486 100.07608:30593 101.042:96251 102.03766:2323042 103.02137:147277 103.04131:40381 103.05506:51303 105.0011:467144 105.07049:117386 106.00422:11288 107.04906:73388 111.02647:25051 112.07571:30476 113.0293:99951 113.06004:38164 117.05665:19657 118.06488:49893 118.08678:35850 120.08085:187201 121.06537:47665 121.08427:13175 128.05235:1937563 129.0365:189459 129.0563:78393 129.06987:30280 130.06438:17176 131.04909:14634 133.06546:18207 134.05949:23870 135.06767:253591 136.07523:23311 146.02765:133879 146.06299:396849 146.09641:432536 147.0473:13737 147.0659:26742 147.09973:16946 159.06662:26672 164.10765:16877 174.05702:1001735 174.08925:90716 175.04242:64969 175.06021:60948 177.05963:14868 191.08054:12279 191.11812:19660 192.03099:20030 192.06886:279670 192.10168:388896 | 22.75750413 |
| Azelate | 0.73 | 171.09764 | HMDB0000784 | 171.09764:62192596 172.10099:12970175 173.10435:12764126 | 55.05506:52578 56.05044:35736 57.07067:13839 58.01451:91130 58.06611:29053 58.99876:14712 67.05518:40863 68.05041:13933 68.98296:47339 69.07066:113400 70.01432:45795 70.06612:52448 72.03002:15973 72.04519:45852 72.08165:15908 76.02479:29217 76.03988:10290 77.99356:9076 79.05473:14752 80.05:11318 81.07111:12013 82.06602:14958 83.06142:15141 83.08635:38321 84.08163:426329 85.06564:13252 85.08499:23334 86.06098:12841 86.09736:180620 86.99328:40767 88.02484:28486 89.07113:36313 93.07013:21297 95.08647:10413 96.03004:145647 97.07646:11090 98.00932:11049 100.02486:36634 100.07612:11607 102.04063:12980 105.0032:15182 108.05087:10013 110.07188:202822 111.04427:10720 112.06113:18010 113.05652:54616 114.04018:389227 114.07605:53688 115.04113:26203 116.05539:9529 118.03572:19350 125.07099:9834 126.07594:18323 128.05513:13701 130.07146:31067 132.05086:101230 132.08684:286098 133.05241:9428 133.08881:31480 139.07159:13378 141.08737:323905 142.08768:30670 143.1035:955774 144.08658:54042 144.1039:106855 146.06633:11270 150.09756:225408 151.09845:16388 154.07106:8751 156.07658:24799 159.09735:233936 160.09915:26505 161.1127:1123902 162.11423:142390 163.1172:10138 169.08102:16148 169.28348:8841 171.09657:2174122 | 4.682345725 |
| Hexadecanedioic acid | 8.912 | 309.20294 | HMDB0000672 | 309.20294:82437192 310.20629:24581468 311.20965:5624153 | 50.63047:14184 52.63464:12959 53.50248:13819 56.40435:14505 57.32672:15872 57.93041:13906 62.11072:12975 67.05522:28709 69.07069:18767 69.74746:13316 97.03116:14893 129.86214:13548 143.07758:16526 165.84415:14911 189.68054:16776 225.9917:15309 230.34552:16805 | 3.302529385 |
| 1-Monopalmitin | 9.41 | 353.26575 | HMDB0245964 | 353.26575:1983256960 354.2691:824154560 355.27246:99228576 | 56.74781:713783 64.54049:594735 66.9543:617904 90.05592:1869193 124.26573:623223 149.32455:672416 157.77512:629565 234.59103:655945 353.26373:18041590 | 18.95906895 |

"PEH-HDH" indicates enzyme hydrolysates of *Perinereis aibuhitensis* with the highest degree of hydrolysis; "P-MRPs" denotes *P. aibuhitensis* Maillard reaction product. FC represents the ratio of the substance's concentration in P-MRPs to its concentration in PEH-HDH.

**Table S2.** Detailed information of differential metabolites between groups

| **ID** | **full names** | **formula** | **HMDB ID** | **KEGG ID** | **Lipidmaps ID** |
| --- | --- | --- | --- | --- | --- |
| U1 | cytidine | C9H13N3O5 | HMDB0000089 | C00475 | - |
| U2 | ureidopropionic acid | C4H8N2O3 | HMDB0000026 | C02642 | - |
| U3 | propionylcarnitine | C10H19NO4 | HMDB0000824 | C03017 | LMFA07070105 |
| U4 | deoxycytidine | C9H13N3O4 | HMDB0000014 | C00881 | - |
| U5 | cytosine | C4H5N3O | HMDB0000630 | C00380 | - |
| U6 | L-Asp | C4H8N2O3 | HMDB0000168 | C00152 | - |
| U7 | 1-18:3-lysoPC | C26H48NO7P | HMDB0010388 | - | LMGP01050038 |
| U8 | LysoPC(20:5(5Z,8Z,11Z,14Z,17Z)/0:0) | C28H48NO7P | HMDB0010397 | C04230 | LMGP01050050 |
| U9 | LysoPC(18:3(6Z,9Z,12Z)/0:0) | C26H48NO7P | HMDB0010387 | C04230 | LMGP01050128 |
| U10 | LysoPC(22:5(4Z,7Z,10Z,13Z,16Z)/0:0) | C30H52NO7P | HMDB0010402 | C04230 | LMGP01050142 |
| U11 | LysoPC(22:5(7Z,10Z,13Z,16Z,19Z)/0:0) | C30H52NO7P | HMDB0010403 | C04230 | LMGP01050143 |
| U12 | LysoPE(0:0/18:3(9Z,12Z,15Z)) | C23H42NO7P | HMDB0011479 | - | LMGP02050043 |
| U13 | LysoPE(0:0/16:1(9Z)) | C21H42NO7P | HMDB0011474 | - | LMGP02050037 |
| U14 | D-glucurono-6,3-lactone | C6H8O6 | HMDB0006355 | C02670 | - |
| U15 | 1-(beta-D-ribofuranosyl)-1,4-dihydronicotinamide | C11H16N2O5 | HMDB0011648 | C15497 | - |
| U16 | acetylcarnitine | C9H17NO4 | HMDB0240773 | C02571 | LMFA07070060 |
| U17 | allolithocholic acid | C24H40O3 | HMDB0000381 | - | LMST04010005 |
| U18 | Pro-Gly | C7H12N2O3 | HMDB0011178 | - | - |
| U19 | Asp-Val | C9H16N2O5 | HMDB0028766 | - | - |
| U20 | Hyp-Asp | C9H15N3O5 | HMDB0028858 | - | - |
| U21 | Lys-Asp-Tyr | C19H28N4O7 | HMDB0304806 | - | - |
| U22 | LysoPE(18:2w6/0:0) | C23H44NO7P | HMDB0011507 | - | LMGP02050011 |
| U23 | LysoPE(0:0/22:5(7Z,10Z,13Z,16Z,19Z)) | C27H46NO7P | HMDB0011495 | - | LMGP02050059 |
| U24 | LysoPC(22:4(7Z,10Z,13Z,16Z)/0:0) | C30H54NO7P | HMDB0010401 | C04230 | LMGP01050124 |
| U25 | LysoPC(14:0/0:0) | C22H46NO7P | HMDB0010379 | - | LMGP01050012 |
| U26 | decanoylcarnitine | C17H33NO4 | HMDB0000651 | C03299 | LMFA07070059 |
| U27 | 1-methyl-2-nonyl-4(1H)-quinolinone | C19H27NO | HMDB0030519 | - | - |
| U28 | castanospermine | C8H15NO4 | HMDB0249700 | C02256 | - |
| U29 | alpha-linolenic acid | C18H30O2 | HMDB0001388 | C06427 | LMFA01030152 |
| U30 | N-stearoyl arginine | C24H48N4O3 | HMDB0241937 | - | LMFA08020136 |
| U31 | N-stearoyl leucine | C24H47NO3 | HMDB0241945 | - | - |
| U32 | N-stearoyl phenylalanine | C27H45NO3 | HMDB0062342 | - | LMFA08020093 |
| U33 | N-myristoyl histidine | C20H35N3O3 | HMDB0242052 | - | - |
| U34 | N-myristoyl tyrosine | C23H37NO4 | HMDB0242062 | - | - |
| U35 | N-docosahexaenoyl histidine | C28H39N3O3 | HMDB0242015 | - | LMFA08020134 |
| U36 | N-docosahexaenoyl serine | C25H37NO4 | HMDB0242021 | - | - |
| U37 | N-eicosapentaenoyl histidine | C26H37N3O3 | HMDB0242072 | - | - |
| U38 | N-oleoyl cysteine | C21H39NO3S | HMDB0241957 | - | - |
| U39 | N-palmitoyl leucine | C22H43NO3 | HMDB0241928 | - | LMFA08020115 |
| U40 | N-palmitoyl arginine | C22H44N4O3 | HMDB0241920 | - | - |
| U41 | N-palmitoyl lysine | C22H44N2O3 | HMDB0241929 | - | - |
| U42 | N-docosahexaenoyl phenylalanine | C31H41NO3 | HMDB0062333 | - | LMFA08020094 |
| U43 | N-docosahexaenoyl lysine | C28H44N2O3 | HMDB0242018 | - | - |
| U44 | N-docosahexaenoyl leucine | C28H43NO3 | HMDB0242017 | - | - |
| D1 | indole-3-carboxaldehyde | C9H7NO | HMDB0029737 | C08493 | - |
| D2 | 4-(2-aminophenyl)-2,4-dioxobutanoic acid | C10H9NO4 | HMDB0000978 | C01252 | - |
| D3 | quinaldic acid | C10H7NO2 | HMDB0000842 | C06325 | - |
| D4 | L-kynurenine | C10H12N2O3 | HMDB0000684 | C00328 | - |
| D5 | indole-3-carboxylic acid | C9H7NO2 | HMDB0003320 | C19837 | - |
| D6 | flavin adenine dinucleotide | C27H33N9O15P2 | HMDB0252293 | C00016 | - |
| D7 | indoleacrylic acid | C11H9NO2 | HMDB0000734 | C21283 | - |
| D8 | nicotinamide adenine dinucleotide (NAD) | C21H27N7O14P2 | - | C00003 | - |
| D9 | 3-methyluridine | C10H14N2O6 | HMDB0004813 | - | - |
| D10 | 3-methyldioxyindole | C9H9NO2 | HMDB0004186 | C05834 | - |
| D11 | glutathione | C10H17N3O6S | HMDB0000125 | C00051 | - |
| D12 | N-arachidonoyl lysine | C26H44N2O3 | HMDB0241999 | - | - |
| D13 | N-arachidonoyl phenylalanine | C29H41NO3 | HMDB0242001 | - | - |
| D14 | N-arachidonoyl methionine | C25H41NO3S | HMDB0242000 | - | - |
| D15 | N-arachidonoyl leucine | C26H43NO3 | HMDB0241998 | - | LMFA08020114 |
| D16 | N-arachidonylglycine | C22H35NO3 | HMDB0005096 | - | LMFA08020003 |
| D17 | N-arachidonoyl serine | C23H37NO4 | HMDB0242003 | - | - |
| D18 | N-palmitoylglycine | C18H35NO3 | HMDB0013034 | - | LMFA08020079 |
| D19 | N-palmitoyl threonine | C20H39NO4 | HMDB0241933 | - | LMFA08020107 |
| D20 | N-palmitoyl valine | C21H41NO3 | HMDB0241935 | - | LMFA08020120 |
| D21 | N-palmitoyl serine | C19H37NO4 | HMDB0241932 | - | LMFA08020101 |
| D22 | N-palmitoyl cysteine | C19H37NO3S | HMDB0241923 | - | - |
| D23 | N-palmitoyl methionine | C21H41NO3S | HMDB0241930 | - | LMFA08020109 |
| D24 | N-linoleoyl threonine | C22H39NO4 | HMDB0241985 | - | - |
| D25 | N-linoleoyl leucine | C24H43NO3 | HMDB0241979 | - | - |
| D26 | N-linoleoyl valine | C23H41NO3 | HMDB0241988 | - | LMFA08020121 |
| D27 | N-linoleoyl lysine | C24H44N2O3 | HMDB0241980 | - | - |
| D28 | N-linoleoyl methionine | C23H41NO3S | HMDB0241981 | - | - |
| D29 | N-linoleoyl glutamine | C23H40N2O4 | HMDB0241975 | - | - |
| D30 | N-docosahexaenoyl alanine | C25H37NO3 | HMDB0242007 | - | - |
| D31 | N-oleoyl glycine | C20H37NO3 | HMDB0241916 | - | LMFA08020082 |
| D32 | N-stearoyl glutamine | C23H44N2O4 | HMDB0241941 | - | LMFA08020130 |
| D33 | delta-decalactone | C10H18O2 | HMDB0037116 | - | LMFA07040028 |
| D34 | quinoline-4-carboxylic acid | C10H7NO2 | HMDB0257047 | C06414 | - |
| D35 | N-acetyl-L-carnosine | C11H16N4O4 | HMDB0012881 | - | - |
| D36 | arachidonoyl ethanolamide | C22H37NO2 | HMDB0004080 | C11695 | LMFA08040056 |
| D37 | 3b-hydroxy-5-cholenoic acid | C24H38O3 | HMDB0000308 | - | LMST04010201 |
| D38 | Ala-Trp | C14H17N3O3 | HMDB0013209 | - | - |
| D39 | Glu-Thr | C9H16N2O6 | HMDB0028829 | - | - |
| D40 | Gly-Glu | C7H13N3O4 | HMDB0028839 | - | - |
| D41 | Asp-Arg | C10H19N5O5 | HMDB0028747 | - | - |
| D42 | Pro-Hyp | C10H16N2O4 | HMDB0006695 | - | - |
| D43 | Arg-Glu | C11H21N5O5 | HMDB0028708 | - | - |
| D44 | Val-Ser | C8H16N2O4 | HMDB0029136 | - | - |
| D45 | Thr-Arg | C10H21N5O4 | HMDB0029055 | - | - |
| D46 | Ile-Thr | C10H18N2O5 | HMDB0028903 | - | - |
| D47 | Leu-Thr | C10H20N2O4 | HMDB0028939 | - | - |
| D48 | Ala-Glu | C8H14N2O5 | HMDB0028686 | C20958 | - |
| D49 | Allopurinol | C5H4N4O | HMDB0014581 | - | - |

**S2. Supplementary figures**

**Figure S1.** Pearson correlation analysis of quality control (QC) samples in positive ion mode. Pairwise Pearson correlation coefficients were calculated based on the relative quantitative values of all detected metabolites among QC samples. Each cell in the heatmap represents the correlation coefficient (*r*) between two QC injections. Darker color indicates a higher correlation.

**Figure S2.** Effects of different test substances on AML12 mouse hepatocytes. (A) Cell viability following ethanol treatment at 0, 31.25, 62.5, 125, 250, and 500 mmol/L. (B) Cell viability following PEH-HDH treatment at 0, 50, 100, 200, 400, and 800 mg/mL. (C) Cell viability following P-MRPs treatment at 0, 50, 100, 200, 400, and 800 mg/mL. (D) ROS levels in cell supernatants. (E) ALT activity in cell supernatants. (F) AST activity in cell supernatants. Ethanol at concentrations ≥250 mmol/L significantly reduced cell viability (*P* < 0.05; A), while neither PEH-HDH nor P-MRPs exhibited significant cytotoxicity at concentrations below 100 mg/mL (*P* ≥ 0.05; B, C). Based on these results, 250 mmol/L ethanol and 100 mg/mL PEH-HDH/P-MRPs were selected for subsequent experiments. Compared with the alcohol-treated group, P-MRP co-incubation significantly reduced ROS levels and attenuated ALT and AST elevation (D-F; *P* < 0.05), with P-MRPs showing greater protective effects than PEH-HDH. Data are presented as mean ± SEM (*n* = 6 per group). Different lowercase letters above bars indicate significant differences (*P* < 0.05, one-way ANOVA with Tukey’s test).
